# Prioritizing evidence-based practices for acute respiratory distress syndrome using digital data: an iterative multi-stakeholder process

**DOI:** 10.1186/s13012-022-01255-y

**Published:** 2022-12-16

**Authors:** Jennifer N. Ervin, Millie R. Dibble, Victor C. Rentes, Michael W. Sjoding, Michelle N. Gong, Catherine L. Hough, Theodore J. Iwashyna, Anne E. Sales

**Affiliations:** 1 Health Sciences, Office of Health Affairs, West Virginia University, Morgantown, WV USA; 2 Institute for Healthcare Policy and Innovation, University of Michigan, Ann Arbor, MI USA; 3 Department of Learning Health Sciences, University of Michigan, Ann Arbor, MI USA; 4 Division of Pulmonary and Critical Care Medicine, Department of Internal Medicine, University of Michigan, Ann Arbor, MI USA; 5 Divisions of Critical Care Medicine and Pulmonary Medicine, Department of Medicine, Montefiore Health System, Albert Einstein College of Medicine, Bronx, NY USA; 6 Division of Pulmonary and Critical Care Medicine, Oregon Health & Science University, Portland, OR USA; 7 Division of Pulmonary and Critical Care, Department of Medicine and Department of Health Policy and Management, Johns Hopkins University, Baltimore, MD USA; 8 Sinclair School of Nursing and Department of Family and Community Medicine, University of Missouri, Columbia, MO USA; 9 VA Center for Clinical Management Research, VA Ann Arbor Healthcare System, Ann Arbor, MI USA

**Keywords:** Implementation science, Prioritization, Evidence-based practices, Clinical quality improvement, Healthcare providers

## Abstract

**Background:**

Evidence-based practices (EBPs) for patients receiving invasive mechanical ventilation vary in the quality of their underlying evidence and ease of implementation.

**Research question:**

How do researchers and clinicians prioritize EBPs to help guide clinical decision-making and focus implementation efforts to improve patient care using existing, validated measures?

**Study design and methods:**

We developed a 4-step rapid method using existing criteria to prioritize EBPs associated with lower mortality and/or shorter duration of invasive mechanical ventilation for patients suffering from acute respiratory failure or acute respiratory distress syndrome. Using different types of data including surveys, we (1) identified relevant EBPs, (2) rated EBPs using the Guideline Implementability Appraisal (GLIA) tool, (3) surveyed practicing ICU clinicians from different hospital systems using a subset of GLIA criteria, and (4) developed metrics to assess EBP performance. In this paper, we describe steps 2 and 3.

**Results:**

In step 2, we prioritized 11 EBPs from an initial list of 30, using surveys and ratings among a small group of clinician researchers. In step 3, 42 clinicians from 8 different hospital systems provided assessments of these 11 EBPs which inform the final step of metric development.

**Interpretation:**

Our prioritization process allowed us to identify 11 EBPs out of a larger group that clinicians perceive is most likely to help optimize invasive mechanical ventilation and improve the outcomes of this vulnerable patient population. While this method was developed in critical care related to adults receiving invasive mechanical ventilation, it is adaptable to other health contexts.

**Supplementary Information:**

The online version contains supplementary material available at 10.1186/s13012-022-01255-y.

Contributions to the literature

- Methods for prioritizing evidence-based practices for implementation in complex clinical care are not well detailed.
- We describe a systematic approach that can be used across multiple settings to conducting essential prioritization, incorporating the views of the research team and clinicians who are the target of implementation activities.
- We also demonstrate the use of a simple approach, radar graphs, to visualize results of multidimensional assessment.

## Introduction

The translational science movement has helped enhance patient care by incorporating clinical research into daily practice. As clinical research evolves, the number of evidence-based practices (EBPs) for a given health problem may also grow. Consequently, deciding which EBPs are most clinically important and feasible to be implemented in care can become burdensome for providers. In addition, it is essential to prioritize EBPs for implementation, given limited resources and time. However, little guidance is provided for this work in implementation process models, in part because the specifics depend on the characteristics of the processes and context in which implementation is being done. To address this need, we used existing criteria to develop a rapid 4-step method for prioritizing EBPs that can be replicated across settings and EBPs. In this report, we outline this method and provide an example of the prioritization process in the context of invasive mechanical ventilation in intensive care unit (ICU) treatment of acute respiratory failure and acute respiratory distress syndrome.

Prior to the COVID pandemic, approximately 200,000 critically ill adults received invasive mechanical ventilation in an ICU for acute respiratory failure and acute respiratory distress syndrome in the USA each year [1–3]. These are vulnerable patient populations—mortality rates remain high at 30–40%, and survivors are at risk for a number of poor outcomes [1–4]. EBPs that improve the outcomes of patients who receive invasive mechanical ventilation are described in multiple guidelines, yet recommendations regarding the care of these patients have not been fully implemented into routine practice [5–8]. Clinicians may find it difficult to choose among the many EBPs supported by reasonable evidence. The team environment of critical care makes this a complex task, as the opinion of a single provider is rarely sufficient. While categorizing the EBPs across complex processes of care is helpful in knowing *when* an EBP might be implemented, yet we lack systematic and replicable processes for helping clinicians decide *which* EBPs to prioritize and implement in these complex care scenarios, where processes overlap and patient care progresses at different rates through the phases of an idealized care continuum. To address this gap, we developed a method using existing and reproducible tools to prioritize EBPs while also recognizing and accounting for the interrelations among EBPs across the care continuum. As made evident by the ongoing COVID epidemic, optimizing invasive mechanical ventilation is of utmost importance, when it is required, but the methods we describe can be used in many similar clinical contexts.

Our objective in this paper is to describe our methods for prioritizing EBPs for the provision of invasive mechanical ventilation for acute respiratory failure/acute respiratory distress syndrome. To illustrate the approach, we report data from a network of 8 hospital systems across the USA in which we developed and used the methods. Overall, our goal is to improve the delivery of care to critically ill adults while describing a scalable method of EBP prioritization that can be used in other settings.

## Study design and methods

Our research team included clinicians and researchers from 4 health systems specializing in pulmonary and critical care medicine, implementation science, learning health systems, and organizational behavior. The larger project was a planning grant from the US National Heart, Lung and Blood Institute of the National Institutes of Health (U01HL143453, Sales and Gong co-PIs), called Digital Implementation Trials in Acute Lung Care (DIGITAL-C), with the ultimate goal to plan a multi-site hybrid type 2 implementation-effectiveness trial of digital implementation strategies. We report abridged methods here; more detailed background and methods are available in Additional file 1.

We engaged in a 4-step multi-method process to evaluate and prioritize EBPs to assist in clinical decision-making and concentrate future implementation efforts. Throughout this process, we focused on EBPs most relevant to and strongly associated with improved clinical outcomes (i.e., shorter duration of mechanical ventilation and/or lower mortality), as identified in previously established guidelines, among patients receiving invasive mechanical ventilation for acute respiratory failure or acute respiratory distress syndrome. In steps 2–4, we also considered the feasibility of using digital data, extracted from electronic health records, to assess EBP performance, rather than processes of human data abstraction.

An overview of our 4-step prioritization process is shown in Table 1. In step 1 (see Ervin et al. [8]), clinician experts from our research team identified key guidelines that included several EBPs, and we searched the literature for related reviews.

**Table 1:** The 4-step prioritization process overview

Step	Definition	Participants	Measures	Data and output
1) Identify potentially relevant EBPs	Compile a list of EBPs that are most relevant to ARF/ARDS and supported by the most robust data	DIGITAL-C research team	Mortality benefit; shorter duration of MV; importance	Initial list of EBPs related to ARF/ARDS
2) Assign ratings to EBPs	Distill the list of EBPs using surveys	DIGITAL-C clinician researchers	GLIA 2.0 instrument	Qualitative and quantitative assessments of EBPs; a distilled list of EBPs based on survey results
3) Frontline clinician panel	Evaluate the distilled list of EBPs from step 2 using surveys	Frontline clinicians	Abbreviated GLIA instrument: measurability; resource intensiveness; source credibility	Quantitative and qualitative evaluation of the distilled list of EBPs
4) Final synthesis	Identify final list of EBPs that will be the focus of implementation efforts	DIGITAL-C research team	Measurability; variability in practice	Highest rated EBPs based on steps 3 & 4

*ARDS* acute respiratory distress syndrome, *ARF* acute respiratory failure, *EBPs* evidence-based practices, *GLIA* guideline implementability appraisal, *MV* invasive mechanical ventilation

Step 1 is reported in our previous paper, in which we describe the 20 EBPs that we collated from the literature review [8]. Step 1 was conducted from July to December 2018. The focus of the current report is on steps 2 and 3.

In step 2, clinician researchers on our team (MWS, MNG, TJI, CLH) rated a list of 26 EBPs generated from step 1 using two Qualtrics surveys. The first survey contained the full set of criteria from the Guideline Implementability Appraisal 2.0 (GLIA) tool, which we show in Table 2 [9].

**Table 2:** Guideline Implementability Appraisal 2.0 (GLIA) variables and definitions

Variable	Definition
Measurability	Endpoints or markers are well identified in this EBP to make it easily measurable and computable in the EMR
Clarity of execution	EBP is clear on how the recommendation should be executed, with “what” and “how” defined, including step-by-step instructions
Decidability	EBP has high clarity as to under what conditions to perform the EBP (e.g., age, gender, clinical findings, laboratory results
Validity	Recommendation highly reflects the intent of the developer and the quality of evidence
Flexibility	The recommendation permits interpretation and allows for alternatives in its execution
Effect on process of care	The recommendation can be carried out without substantial disruption of current workflow or significant increased need for resources
Novelty/innovation	The recommendation proposes behaviors considered new and unconventional by clinicians (or patients)
Resource intensiveness	Whether the EBP is resource intensive
Clarity of target population	The guideline clearly defines the target patient population
Source credibility	The organizations and authors who developed the guideline have credibility with the intended audience of the guideline
Consistency	The recommendation is consistent among other authors in the literature and your understanding of evidence-based practice

*EBP* evidence-based practice, *EMR* electronic health record

**Fig. 1:**
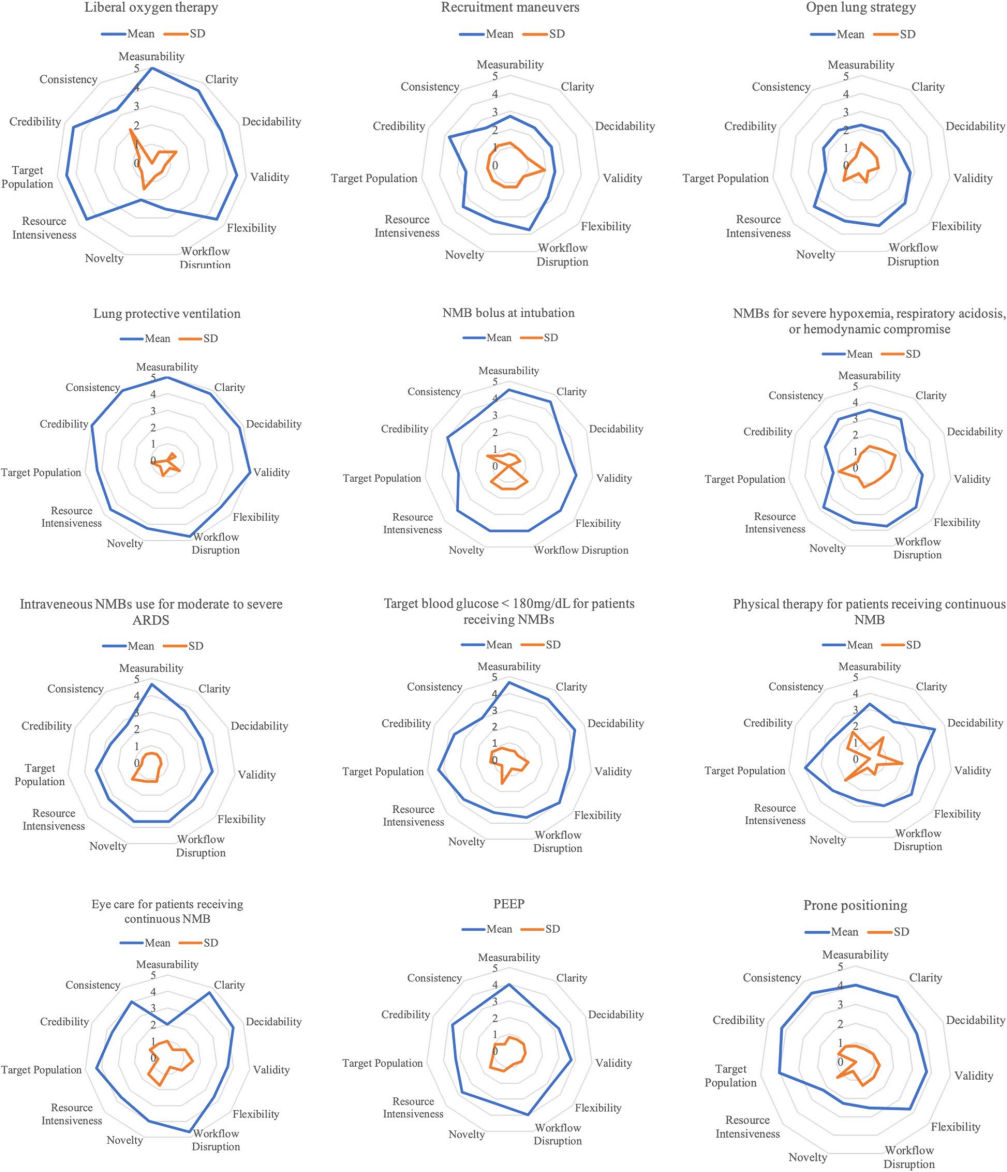
Radar graphs for phase 1 EBPs

**Fig. 2:**
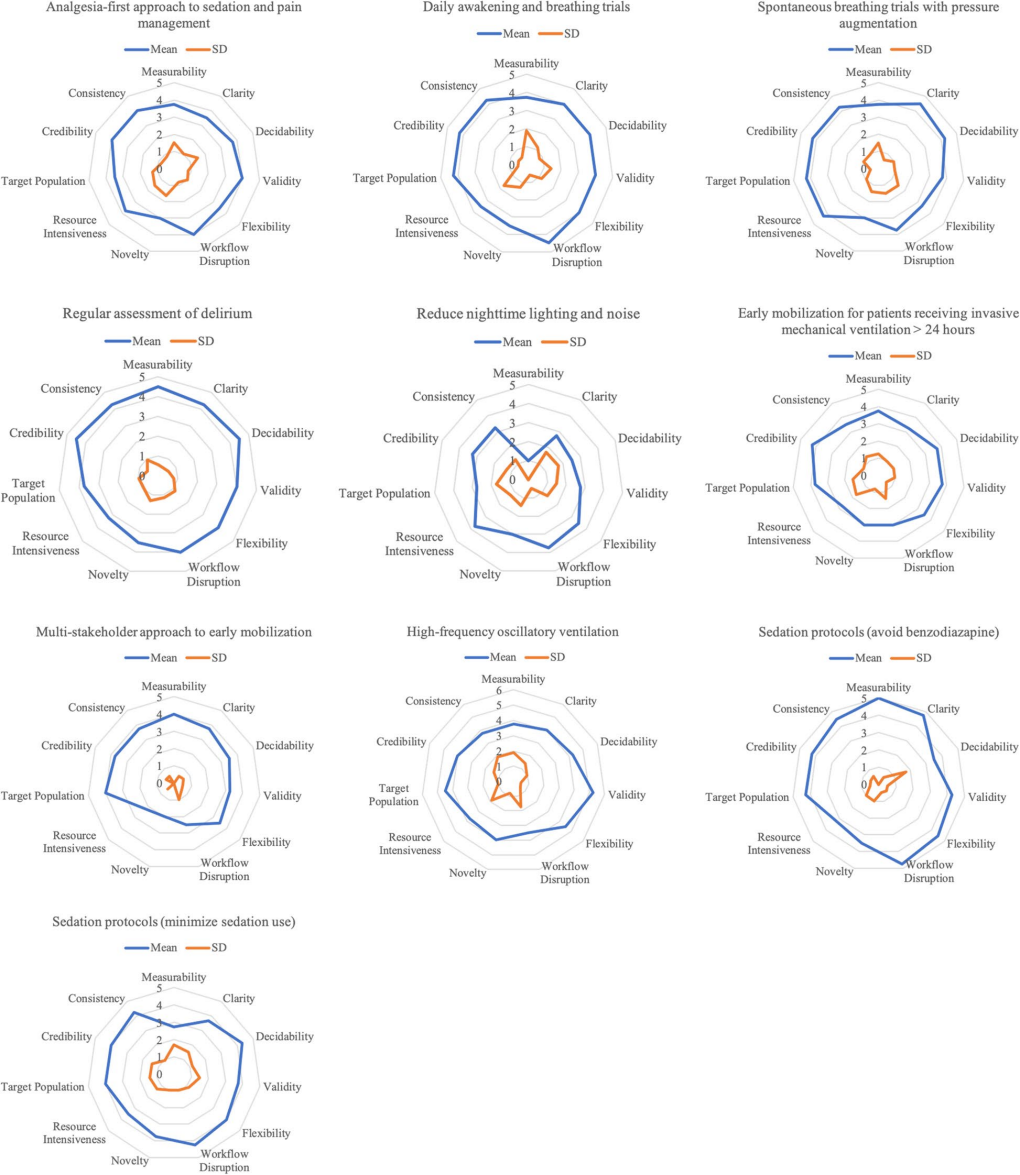
Radar graphs for phase 2 EBPs

**Fig. 3:**
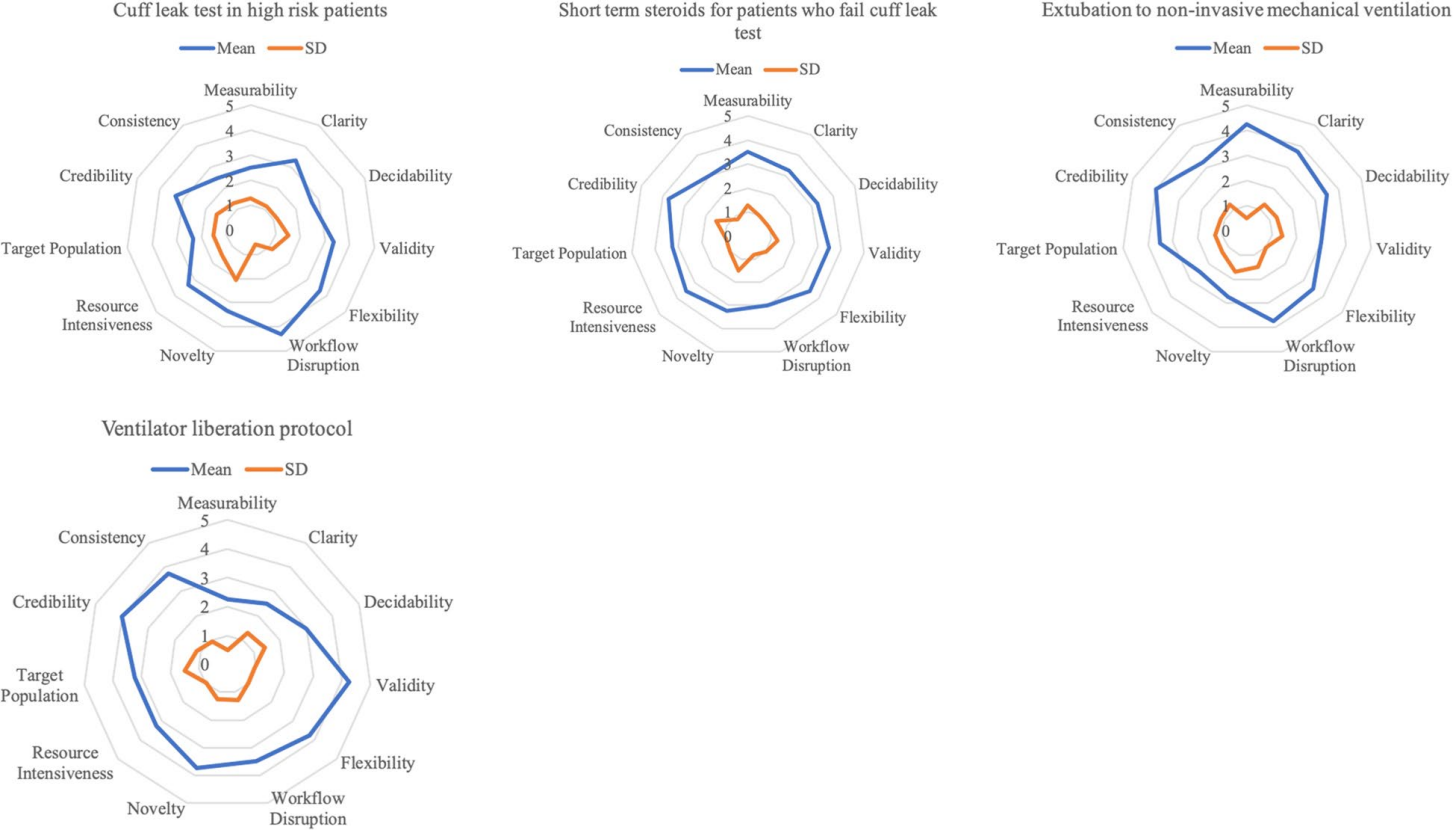
Radar graphs for phase 3 EBPs

In this and subsequent steps, we used radar graphs (Figs. 1, 2 and 3) to assess responses across all of the GLIA dimensions concurrently. To construct these graphs, we averaged the responses and plotted them on the 11 axes of the GLIA dimensions, using Microsoft Excel. At this point, following discussion focused on the radar graphs, we removed a total of 15 EBPs from the list, leaving 11. Our primary criteria focused on measurability, resource intensiveness, and source credibility. We conducted step 2 in February-May 2019.

In step 3, frontline clinicians from 8 participating hospital systems evaluated the distilled list of 11 EBPs produced from step 2 using a much-reduced survey instrument. We reduced the survey to 3 GLIA elements (measurability, resource intensiveness, and source credibility) based on the experience of the team clinicians who engaged in the first 2 rounds of surveys, who felt that the full GLIA was very burdensome. We conducted these surveys in June-September 2019.

We surveyed clinicians directly involved in caring for patients receiving invasive mechanical ventilation, including attending physicians, house staff, nurse managers, registered nurses, and respiratory therapists. We used Qualtrics as the platform for the survey. Clinicians were asked whether we should include the EBPs in the final list for implementation (yes; no; maybe) and then rated the EBPs on 3 GLIA criteria: measurability, resource intensiveness, and source credibility. These three criteria were selected based on the specific needs of this project, which included the ability to use electronic medical record data to extract data for measurement, feasibility which we operationalized as “resource intensiveness,” and whether the recommendation came from a credible source. These are modified from the original GLIA terms and were selected based on input from the clinician members of the research team. We note that we used the term “source credibility” rather than “evidence validity” to reduce the burden to clinicians of fully assessing the validity of the evidence for each recommendation. In future work, we recommend that all the GLIA questions be considered for prioritization, depending on the groups involved in assessing recommendations. Descriptive statistics were calculated in Microsoft Excel; missing data were omitted using pairwise deletion.

The 4th and final step, not addressed in this paper, was to use all of the available data to generate the final list of EBPs to focus future implementation efforts. This step required developing metrics for each of the included EPBs using digitally extracted data from electronic health records, which we will report in subsequent papers. We placed heavy emphasis on measurability and variability in practice within and across ICUs and health systems.

This study was deemed exempt from human subject’s oversight by IRBMED at the University of Michigan. Data were gathered throughout 2019.

## Results

### Step 1: Identifying EBPs

Our process of initially identifying relevant EBPs is described in Ervin et al. [8], and involved a review of reviews to identify EBPs along the full continuum (three phases) of invasive mechanical ventilation care, corresponding to specific processes of care.

### Step 2: Rating EBPs

We present findings from the first survey visually as radar graphs (see Figs. 1, 2 and 3) and in Additional file 2—tabular results of the clinician researcher review. The radar graphs show the degree of variation across the ratings for the EBPs. The graphs showing a fully rounded circle were rated most highly across all dimensions of the GLIA, while those with jagged circles, with some dimensions scoring a 1 or 2 rather than a 4 or 5, demonstrate that some GLIA elements were rated highly while others were not. For example, in Fig. 1, lung-protective ventilation was rated high on all domains with little variability, whereas the use of recruitment maneuvers was rated mid to low on most domains with greater variability.

The second survey completed by clinician researchers contained the 26 EBPs from step 1, as well as 4 additional EBPs identified by clinician researchers after the first survey: protocol-based pain assessment and management, conservative fluid management, daily awakening trials, extubation to high-flow nasal cannula. Findings from the second survey supported the prioritization of 11 EBPs (Additional file 2).

The prioritized EBPs from step 2 represent processes across the continuum of invasive mechanical ventilation care; the 2 EBPs associated with escalation of care and initiation of invasive mechanical ventilation were lung-protective ventilation [4] and prone position [4]; 6 EBPs reducing complications included an analgesia-first approach to sedation [10], protocol-based pain assessment and management [11], conservative fluid management [12], daily awakening and breathing trials [13], early mobilization [11], and sedation protocols [11]; and the 3 EBPs associated with de-escalation of care and post-extubation recovery included use of a ventilator liberation protocol [14], extubation to noninvasive ventilation [15], and extubation to high-flow nasal cannula [16].

### Step 3: Engage stakeholders beyond the research team

Forty-two ICU clinicians from 8 integrated health systems across the USA responded to a survey, rating the 11 EBPs identified in step 2 using a shortened survey instrument (Additional file 3 contains all survey instruments). As reported in Table 3, lung-protective ventilation, prone positioning, and sedation protocols were rated the highest in measurability. Early mobilization, prone positioning, and extubation to noninvasive ventilation were rated as the most resource intensive. Lung-protective ventilation, paired spontaneous awakening and breathing trials, and prone positioning were rated the highest in source credibility.

**Table 3:** Tabular results from the frontline clinician panel

Phase of care	EBP	Source	Definition	Include in final list			Measurable	Resource intensive	Source credibility
				Yes	No	Maybe	M (SD)	M (SD)	M (SD)
1	Lung-protective ventilation	Fan et al. (2017) [4]	Mechanical ventilation using lower tidal volumes (4–8 ml/kg predicted body weight) and lower inspiratory pressures (plateau pressure < 30 cm H2O) for adult patients with ARDS	39 (97.5%)	0 (0.0%)	1 (2.5%)	4.79 (0.47)	3.02 (1.17)	4.63 (0.66)
1	Prone positioning	Fan et al. (2017) [4]	Prone positioning for more than 12 h/day for adult patients with severe ARDS	28 (75.7%)	2 (5.4%)	7 (18.9%)	4.54 (0.68)	4.08 (1.27)	4.32 (0.78)
2	Analgesia-first approach to sedation	Strom (2010) [10]	Strategy to keep the amount and duration of sedation to a minimum	25 (65.8%)	4 (10.5%)	9 (23.7%)	3.53 (1.18)	3.47 (1.16)	3.89 (0.79)
2	Protocol-based pain assessment and management	Devlin et al. (2018) [11]	Routine use of an assessment-driven, protocol-based, stepwise approach for pain and sedation management in critically ill adults. Pain should be treated before a sedative agent is considered	28 (73.7%)	5 (13.2%)	5 (13.2%)	3.66 (1.24)	3.42 (1.29)	3.97 (0.87)
2	Conservative fluid management	Semler et al. (2016) [12]	Conservative fluid management.	24 (61.5%)	4 (10.3%)	11 (28.2%)	3.49 (1.19)	3.26 (1.19)	3.78 (0.85)
2	Daily awakening and breathing trials	Girard et al. (2008) [13]	Use of daily sedative interruption/nurse-protocolized sedation to achieve and maintain a light level of sedation	32 (84.2%)	0 (0.0%)	6 (15.8%)	4.13 (0.92)	3.41 (1.16)	4.35 (0.68)
2	Early mobilization	Devlin et al. (2018) [11]	For acutely hospitalized patients who have been mechanically ventilated for > 24 h, suggested protocolized rehabilitation directed toward early mobilization	32 (82.1%)	1 (2.6%)	6 (15.4%)	4.08 (0.84)	4.21 (0.98)	4.19 (0.70)
2	Sedation protocols	Devlin et al. (2018) [11]	Using either propofol or dexmedetomidine is preferred over benzodiazepines for sedation in critically ill, mechanically ventilated adults	35 (89.7%)	1 (2.6%)	3 (7.7%)	4.49 (0.79)	3.13 (1.36)	4.18 (0.93)
3	Ventilator liberation protocol	Schmidt et al. (2017) [14]	Manage acutely hospitalized adults who have been MV > 24 h with a ventilator liberation protocol (designed to reduce variation in practice)	25 (65.8%)	3 (7.9%)	10 (26.3%)	4.03 (1.03)	3.58 (1.18)	4.15 (0.93)
3	Extubation to preventive noninvasive ventilation	Ouellette et al. (2017) [15]	Extubation to preventive noninvasive ventilation (NIV)	11 (29.0%)	16 (42.1%)	11 (29.0%)	3.80 (1.01)	3.72 (1.03)	3.60 (0.95)
3	Extubation to high-flow nasal cannula	Hernandez (2016) [16]	Extubation to high-flow nasal cannula	10 (27.0%)	11 (29.7%)	16 (43.2%)	4.00 (0.82)	3.46 (1.04)	3.50 (0.93)

### Step 4: Final selection through metric creation

We will report the final synthesis methods and results in future reports. In this step, we extracted electronic health record data from 6 of the 8 sites participating in this study to develop performance metrics for the prioritized EPBs.

## Discussion

In this paper, we present a rapid systematic method for prioritizing EBPs using previously established criteria. We developed this process and present invasive mechanical ventilation as an example. A similar multistep process could be used for any discrete clinical processes, especially complex clinical care processes. Prioritization is an essential step for implementation in complex interventions, described in many implementation process models [17–19] although typically with little detail. One approach uses conjoint analysis, which is valuable but quite burdensome and time-consuming to conduct [20]. Other approaches, such as modified Delphi techniques [21, 22], use relatively unstructured brainstorming and selection by varying groups involved in the implementation processes. Steps 2 and 3 of the prioritization process that we describe in this paper, constituting the most direct components of prioritization, took about 3 months to complete. Use of surveys and the GLIA criteria, as well as assessment of clinical importance, facilitated this work.

The literature on implementation in healthcare emphasizes the importance of engaging stakeholders throughout the process of implementation. The process we describe in this paper is feasible and can be used in many settings and provides a method of engaging and obtaining input from a wide range of clinicians (step 3), which is often very difficult in most implementation projects across multiple settings. While interviews and/or observation yield richer data, particularly about thinking underlying responses to criteria, obtaining a broad assessment of criteria is important. Even in high acuity settings, where clinicians often have little time and energy for engaging with researchers and others, a simple survey like the one we used in step 3 can get their essential input. In future work, implementing the final set of EBPs, we will inform clinicians about how EBPs were selected and ultimately prioritized.

We note that despite the importance of a criterion-based approach (the GLIA 2.0 instrument), clinicians on our team found our initial survey to be excessively burdensome, despite their high motivation to respond. Based on their input, we reduced the survey from eleven dimensions to the three that clinician members of the team deemed most important. Even when we administered the survey to the clinicians on the research team, we only used the main dimensions rather than the full GLIA instrument, which is intended to address implementability of guideline recommendations, a more extensive application than ours in this work. This may be important to revisit for other applications of this method, where different GLIA criteria may be relevant.

We detail time and effort required in using this approach in Additional file 1. In steps 2 and 3, we found radar graphs to be particularly helpful when comparing a large number of EBPs on all 11 GLIA criteria. They provide a visual representation that is easier to assimilate than tabular representation of the results, in part by providing easy visuals of variability across criteria and across EBPs. Using these, as well as surveys and established criteria, out of a list of 30, we were able to identify 11 EBPs that met the greatest number of criteria and represented the continuum of care, from intubation to liberation, for patients receiving invasive mechanical ventilation. We note that in future work, we will include clinical importance as a criterion throughout the prioritization process.

Finally, site liaisons and champions have been critical to the success of this work to date. In externally driven work, these champions have helped with buy-in, have coordinated and participated in interviews identifying barriers and facilitators to the prioritized EBPs, and helped identify digital metrics. This work is not possible without engagement and support of these key stakeholders. Engaging them in the discussions and in the review of findings from the surveys has been instrumental in ensuring that our prioritization decisions are based in sites that represent different contexts.

### Limitations

The evidence base in critical care medicine is constantly evolving. Large randomized controlled studies are published each year that change current guidelines and recommendations. Information about how COVID affects patients has already led to changes in the strength of some evidence related to invasive mechanical ventilation care. Therefore, it will be necessary to review and re-prioritize EBPs periodically, to ensure that the most up-to-date evidence is guiding medical decision-making and implementation efforts.

With regard to the prioritization process itself, we found that throughout steps 2 and 3, study participants, as well as members of our research team, were anchoring judgments of measurability based on their own experience and awareness of data. This is not a limitation per se; however, it is important to be aware of individual- versus unit- and health system-level variability.

Our primary purpose in this study is to describe a rapid method of eliciting priorities from clinicians who will be affected by implementation processes. We used pragmatic approaches, such as distributing surveys through site champions, not conducting inference testing to assess statistical significance of agreement among raters, or using the more understandable mean rather than median in creating the radar graphs, for example. Adding levels of rigor to what is often necessarily highly pragmatic work [23], preparing for rigorous implementation, adds cost and complexity to already complex preparatory work.

## Conclusions

Our research team prioritized 11 EBPs that are supported by quality evidence and are feasible to implement but not yet fully implemented in routine practice. Next steps include the development and validation of performance metrics for these EPBs from digital data and the analysis of interview data to identify barriers and facilitators to specific EBPs in order to guide development of implementation interventions to promote evidence-driven care for this vulnerable patient population.

## Supplementary Information

**Additional file 1.** Additional background and methods details.

**Additional file 2.** Tabular results from Step 2 survey of research clinical team.

**Additional file 3.** Combined surveys for Steps 2 and 3.

## Data Availability

Data are available by contacting the senior author, Anne Sales.

## Abbreviations

EBP: Evidence-based practices
ICU: Intensive care unit
