# Supplementary material for: Prioritizing evidence-based practices for acute respiratory distress syndrome using digital data: an iterative multi-stakeholder process
Source: Implement Sci. 2022 Dec 16;17:82. doi: 10.1186/s13012-022-01255-y (PMC9756680; doi:10.1186/s13012-022-01255-y)
Supplement: Supplementary file 1 — Additional file 1. Additional background and methods details. [file 13012_2022_1255_MOESM1_ESM.docx]

**Additional background information on invasive mechanical ventilation in acute respiratory failure and acute respiratory distress syndrome**

Prior to the COVID pandemic, approximately 200,000 critically ill adults received IMV in an ICU for ARF and ARDS in the US each year.[1–3] These are vulnerable patient populations—mortality rates remain high at 30-40%, and survivors are at risk for a number of poor outcomes.[1–4] EBPs that improve the outcomes of patients who receive IMV are described in multiple guidelines, yet recommendations regarding the care of these patients have not been fully implemented into routine practice.[5–8] Clinicians may find it difficult to choose among the many EBPs supported by reasonable evidence. The team environment of critical care makes this a complex task, as the opinion of a single provider is rarely sufficient. This situation is made more acute with COVID and associated disease, which has increased the need for IMV even among relatively healthy individuals.[5] As is true with many health interventions, IMV requires multiple steps to be taken to achieve the desired treatment effect. For example, in this context, the care process for providing IMV to treat ARF/ARDS can be described as a continuum, which we used to categorize EBPs as *early interventions* to be used at or soon after intubation, those that contribute to the *de-escalation of interventions*, to *extubation and* *discontinuation of IMV*.[8] The three phases constitute a very high level process map for invasive mechanical ventilation. While categorizing the EBPs across such a care continuum is helpful in knowing *when* an EBP might be implemented, as yet we lack systematic and replicable processes for helping clinicians decide *which* EBPs to prioritize and implement in these complex care scenarios, where processes overlap, and patient care progresses at different rates through the phases of an idealized care continuum. To address this gap, we developed a method using existing and reproducible tools to prioritize EBPs, while also recognizing and accounting for the interrelations among EBPs across the care continuum. As made evident by the ongoing COVID epidemic, optimizing IMV is of utmost importance, when it is required, but the methods we describe can be used in many similar clinical contexts.

**Further details on Methods**

*Overview*:

Our research team included clinicians and researchers from 4 health systems specializing in pulmonary and critical care medicine, implementation science, learning health systems, and organizational behavior. Specifically, the team included critical care physicians, a registered nurse, and health services researchers. We consulted extensively with respiratory therapists as part of a larger project. The larger project was a planning grant from the US National Heart, Lung and Blood Institute of the National Institutes of Health (U01HL143453, Sales and Gong co-PIs), called Digital Implementation Trials in Acute Lung Care (DIGITAL-C), with the ultimate goal to plan a multi-site hybrid type 2 implementation-effectiveness trial of digital implementation strategies.

In Step 1, clinician experts from our research team identified key guidelines that included several EBPs. Other research team members searched the literature and identified additional EBPs from systematic reviews and meta-analyses that were not yet incorporated into the current guidelines and recommendations (for results of that search see Ervin et al.[8]). We focused the search on addressing the continuum of care from intubation to extubation, depicted in our published review of reviews[8], describing the three phases in the continuum: Phase 1, intubation and escalation of care; Phase 2, maintenance and early de-escalation; and Phase 3, de-escalation and extubation. Despite depicting these as relatively linear phases, we stress that the phases do overlap for many patients, and that implementation of EBPs across the full continuum of care is a complex endeavor.

Many of the EBPs we initially identified in Phase 1 focused on optimizing ventilator settings, particularly in ARDS—such as guidelines for positive end-expiratory pressure and lung protective ventilation. EBPs in Phase 2 focused on facilitating timely extubation by beginning de-escalation. Many of these Phase 2 EBPs fit with the Society of Critical Care Medicine’s (2020) ICU Liberation Bundle, including recommendations for daily paired spontaneous awakening trials and breathing trials, and early mobility.[9] Phase 3 represented EBPs that prepare the patient for extubation (e.g., the use of a ventilator liberation protocol; cuff leak tests) as well as guidelines for post-extubation care, such as extubation to heated high-flow nasal cannula or noninvasive mechanical ventilation.

*Step 2:*

Team members rated each EBP for: measurability; clarity of execution; decidability; validity; flexibility; effect on process of care; novelty/innovation; resource intensiveness; clarity of target population; author credibility; consistency (see Table 2). Responses were captured on 1-5 Likert-type scales anchored by *Strongly Disagree* and *Strongly Agree*. Open text boxes allowed clinicians to write in any additional feedback they had about each EBP. The clinician researchers then completed a second survey, which focused solely on the clinical importance of the EBPs, a dimension that was not included in the initial GLIA survey. Some additional EBPs were added at this stage, based on clinician input. Participants were prompted to consider whether each EBP should be included in the next stage of prioritization (Yes; No; Maybe). Open text boxes were provided so that participants could indicate whether they thought certain EBPs were interdependent. The full research team discussed the data and used the findings to initially prioritize those EBPs with the highest ratings and greatest consensus among the team.

*Step 3:*

We surveyed clinicians directly involved in caring for patients receiving IMV, including attending physicians, house staff, nurse managers, registered nurses, and respiratory therapists from research team members’ home institutions. Site champions, identified as part of the overall project, forwarded an email with the survey link to clinicians in their sites. We used Qualtrics as the platform for the survey. Clinicians were asked whether we should include the EBPs in the final list for implementation (Yes; No; Maybe), and then rated the EBPs on 3 GLIA criteria: measurability; resource intensiveness; source credibility. We focused on these 3 criteria because they pose some of the greatest potential barriers to implementation—inability to monitor performance; the effort is too great to implement the EBP; lack of trust in the evidence base or the source of the EBP guidelines. Responses to these GLIA criteria were captured on 1-5 scales anchored by Strongly Disagree and Strongly Agree. We also provided open text boxes that allowed clinicians to write in any additional feedback they had about each EBP. Descriptive statistics were calculated in Microsoft Excel; missing data were omitted using pairwise deletion.

*Estimated effort for this method*

An important question is whether there is value in adding the effort of a survey to other approaches, often quite *ad hoc*, to prioritizing a large number of possible EBPs or recommendations for practice for implementation, as we have done in this work. First, as we noted above, the amount of additional effort for the short, web-based survey we fielded, particularly through site champions as we did, was not large. We estimate that it took about 3 hours to develop the final survey in Qualtrics, and once it was developed, no more than an hour of project staff time to send it to our site champions. The amount of time it took for them to distribute to clinicians they selected was likely variable, and probably depended on whether they sent it to an established listserv in critical care, as was done in most sites. Downloading and analyzing the data descriptively, as we did, took several hours, probably on the order of 10-15 hours altogether. We spent about 3 hours discussing it, over the course of several meetings. As we note earlier, this is work that needs to be done for most complex implementation projects, and is not much more intensive or time-consuming than other approaches used by researchers in our team.

References

1. Wunsch H, Linde-Zwirble WT, Angus DC, Hartman ME, Milbrandt EB, Kahn JM. The epidemiology of mechanical ventilation use in the United States. Crit Care Med. Ovid Technologies (Wolters Kluwer Health); 2010;38:1947–53.

2. Rubenfeld GD, Caldwell E, Peabody E, Weaver J, Martin DP, Neff M, et al. Incidence and outcomes of acute lung injury. N Engl J Med. Massachusetts Medical Society; 2005;353:1685–93.

3. Stefan MS, Shieh M-S, Pekow PS, Rothberg MB, Steingrub JS, Lagu T, et al. Epidemiology and outcomes of acute respiratory failure in the United States, 2001 to 2009: a national survey. J Hosp Med. Wiley; 2013;8:76–82.

4. Fan E, Del Sorbo L, Goligher EC, Hodgson CL, Munshi L, Walkey AJ, et al. An official American thoracic society/European society of intensive care medicine/society of critical care medicine clinical practice guideline: Mechanical ventilation in adult patients with acute respiratory distress syndrome. Am J Respir Crit Care Med. American Thoracic Society; 2017;195:1253–63.

5. Phua J, Weng L, Ling L, Egi M, Lim C-M, Divatia JV, et al. Intensive care management of coronavirus disease 2019 (COVID-19): challenges and recommendations. Lancet Respir Med. Elsevier BV; 2020;8:506–17.

6. Weiss CH. Why do we fail to deliver evidence-based practice in critical care medicine? Curr Opin Crit Care. 2017;23:400–5.

7. Kahn JM. Bringing implementation science to the intensive care unit. Curr Opin Crit Care. 2017;23:398–9.

8. Ervin J, Rentes VC, Dibble E, Sjoding MW, Hough CTL, Iwashyna TJ, et al. Evidence Based Practices in the Continuum of Care for Mechanically Ventilated Patients: A Review of Reviews. C46 CRITICAL CARE: ACUTE RESPIRATORY FAILURE AND MECHANICAL VENTILATION. American Thoracic Society; 2020. p. A5235–A5235.

9. Society of Critical Care Medicine. ICU Liberation Bundle [Internet]. [cited 2022 Aug 29]. Available from: https://www.sccm.org/iculiberation
