# Supplementary material for: Prioritizing evidence-based practices for acute respiratory distress syndrome using digital data: an iterative multi-stakeholder process
Source: Implement Sci. 2022 Dec 16;17:82. doi: 10.1186/s13012-022-01255-y (PMC9756680; doi:10.1186/s13012-022-01255-y)
Supplement: Supplementary file 2 — Additional file 2. Tabular results from Step 2 survey of research clinical team. [file 13012_2022_1255_MOESM2_ESM.docx]

| Phase of Care | EBP | Definition | Include in Next List* | | | Qualitative Comments |
| --- | --- | --- | --- | --- | --- | --- |
|  |  |  | Yes | No | Maybe |  |
| 1 | Liberal oxygen therapy | Recommend against liberal oxygen therapy in acutely ill adults (aged > 18 years) defined as saturation of peripheral oxygen (Sp02) above range of 94-96%. | 0  (0.0%) | 1  (33.3%) | 2 (66.6%) | MD1: Personally I feel yes but others might differ and there are ongoing trials.  MD3: Not proven, although what I do. |
| 1 | Recruitment maneuvers | Recruitment maneuvers for adult patients with moderate or severe ARDS | 0  (0.0%) | 3 (100%) | 0  (0.0%) | MD2: Not very compelling evidence |
| 1 | Open lung strategy | Open lung strategy during mechanical ventilation for patients with ARDS. | 0  (0.0%) | 3 (100%) | 0  (0.0%) | MD3: Undefined. |
| 1 | Lung protective ventilation | Mechanical ventilation using lower tidal volumes (4–8 ml/kg predicted body weight) and lower inspiratory pressures (plateau pressure < 30 cm H2O) for adult patients with ARDS | 3 (100%) | 0  (0.0%) | 0  (0.0%) |  |
| 1 | NMB bolus at intubation | For rapid sequence intubation, a single intravenous bolus of a short-acting curare such as succinylcholine is recommended to permit transient full relaxation of the jaw and larynx muscles, facilitating the introduction of the tracheal tube. | 1 (33.3%) | 2 (66.6%) | 0  (0.0%) | MD1: Many other studies show that it is not always needed. More common in ED intubation.  MD2: I think initial intubation is outside of the scope of this project. |
| 1 | NMBs for severe hypoxemia, respiratory acidosis, or hemodynamic compromise | Use/trial of a neuromuscular blocking agent in life-threatening situations associated with profound hypoxemia, respiratory acidosis, or hemodynamic compromise. | 2 (66.6%) | 0  (0.0%) | 0  (0.0%) | MD1: Can be a useful rescue therapy and leave it to team to judge what is considered profound enough.  MD2: Not sure what makes this an “evidence-based practice.” |
| 1 | Intravenous NMB for moderate to severe ARDS | Neuromuscular blocking agent administered by continuous intravenous infusion early in the course of acute respiratory distress syndrome for patients with a Pa02/Fi02 less than 150. | 0  (0.0%) | 3 (100%) | 0  (0.0%) | MD1: Upcoming trial results show no benefit.  MD3: Cough, cough |
| 1 | Target blood glucose < 180/dL for patients receiving MV | Target blood glucose level for less than 180 mg/dL in patients receiving neuromuscular blocking agents. | 1 (33.3%) | 2 (66.6%) | 0  (0.0%) | MD1: Again this is dependent on NMB. |
| 1 | Physical therapy for patients receiving continuous NMB | Patients receiving a continuous infusion of neuromuscular blocking agent should receive a structured physiotherapy regiment. | 1 (33.3%) | 2 (66.6%) | 0  (0.0%) | MD1: Mobilization should just happen in everyone regardless of NMB.  MD2: I have no idea what this means- positioning to avoid foot drop? Can’t exactly participate in therapy while sedated/paralyzed! |
| 1 | Eye care for patients receiving continuous NMB | Scheduled eye care that includes lubricating drops or gel and eyelid closure for patients receiving continuous neuromuscular blockading agents. | 1 (33.3%) | 1 (33.3%) | 1 (33.3%) | MD1: This is only if using NMB and I am not convinced that we should promote NMB.  MD2: Doesn’t feel like a stand-alone element, rather, part of a NMB approach. |
| 1 | PEEP | Higher PEEP with moderate or severe ARDS | 1 (33.3%) | 0  (0.0%) | 2 (66.6%) | MD1: Conditional recommendations so would support conditions by which this is recommended (i.e., plateau pressure < 30 for moderate to severe ARDS only). |
| 1 | Prone Positioning | Prone positioning for more than 12 hours/day for adult patients with severe ARDS | 2 (66.6%) | 1 (33.3%) | 0  (0.0%) | MD3: Just don’t buy it. Fight me. |
| 2 | Analgesia-first approach to sedation | Analgesia-first approach. | 3 (100%) | 0  (0.0%) | 0  (0.0%) | MD1: Less data on this but I feel like this is important and easy |
| 2 | Protocol-based pain assessment and management | Routine use of an assessment-driven, protocol-based, stepwise approach for pain and sedation management in critically ill adults. Pain should be treated before a sedative agent is considered. | 3 (100%) | 0  (0.0%) | 0  (0.0%) | MD1: Need to also link to sedation protocol, as pain is hard to assess in mechanically ventilated patients and often leads to unnecessary sedation.  MD2: See above—pain and sedation management certainly need to be a part of what we are creating. |
| 2 | Conservative fluid management | Conservative fluid management. | 3 (100%) | 0  (0.0%) | 0  (0.0%) | MD1: Only for ARDS and not in shock. |
| 2 | Daily awakening trial | Use of daily sedative interruption or nurse-protocolized sedation to achieve and maintain a light level of sedation. | 3 (100%) | 0  (0.0%) | 0  (0.0%) | MD1: Consider as part of the protocol but having a protocol aimed at minimizing unnecessary sedation is more important than specifically using daily awakening as the treatment. Regardless daily breathing trial is needed but it should be coupled with sedation protocol to target minimal sedation and awake patients during a breathing trial. This has more to do with coordination of care and should be bundled together.  MD2: In combination with sedation protocols and choice of agents. I think this project could really innovate a new approach for defining high quality components of sedation practice. |
| 2 | Daily awakening and breathing trials | Use of daily sedative interruption/nurse-protocolized sedation to achieve and maintain a light level of sedation | 3 (100%) | 0  (0.0%) | 0  (0.0%) | MD1: Again this relates to coordination and implementation and should be linked to sedation protocols. |
| 2 | Spontaneous breathing trial with pressure augmentation | SBT conducted with inspiratory pressure augmentation (5-8 cm H2O, with pressure support ventilation, automatic tube compensation) rather than without (T-piece or CPAP). | 2 (66.6%) | 0  (0.0%) | 1 (33.3%) | MD1: This is not related to pain. It is the breathing trial linked to minimizing sedation.  MD2: The incremental benefit is not totally clear- could be an element of SBT item but not stand alone.  MD3: Honestly, any SBT. |
| 2 | Regular assessment of delirium | Regular assessment of delirium using validated screening tools for critically ill adults. | 2 (66.6%) | 0  (0.0%) | 1 (33.3%) | MD1: Some would argue that assessment of delirium is not needed and what is needed is uniform non-pharmacologic prevention and treatment for delirium in all patients. |
| 2 | Reduce nighttime lighting and noise | Using noise and light reduction strategies to improve sleep in critically ill adults. | 2 (66.6%) | 0  (0.0%) | 1 (33.3%) | MD2: Data are not strong (and SUPER hard to measure).  MD3: Unassessable. |
| 2 | Early mobilization for patients receiving MV  > 24 hours | For acutely hospitalized patients who have been mechanically ventilated for > 24 hours, suggested protocolized rehabilitation directed toward early mobilization. | 3 (100%) | 0  (0.0%) | 0  (0.0%) | MD1: Same as prior but I am very okay with limiting this for patients on the vent for > 24 hours.  MD2: In some form. |
| 2 | Multi-stakeholder approach to early mobility | Performing rehabilitation or mobilization in critically ill adults with mobility protocols executed by nurses and PTs with family involvement. | 2 (66.6%) | 0  (0.0%) | 1 (33.3%) | MD1: Yes for mobilization protocols but no for PT and family involvement. This is one of multi-layer recommendations that are hard to fully implement. What if in some institutions, PT is not available but nurses are invested and want to do it? Also what if family does not want to get involved? The key rec here is needed for rehabilitation and mobility.  MD2: Although not necessarily as written in the recommendation. I would include avoiding bed rest.  MD3: Is an ongoing trial… |
| 2 | High-frequency oscillatory ventilation | Do not use high-frequency oscillatory ventilation in patients with moderate or severe ARDS. Is not associated with a mortality benefit, and may even be harmful in comparison to ventilation with low tidal volumes and higher levels of PEEP. | 1 (33.3%) | 2 (66.6%) | 0  (0.0%) | MD1: Few sites use this so even fi strong recommendation, it is not really relevant for many sites.  MD2: I don’t think this is used at any of the participating sites.  MD3: Role in rescue |
| 2 | Sedation protocols (avoid benzodiazepine) | Using either propofol or dexmedetomidine is preferred over benzodiazepines for sedation in critically ill, mechanically ventilated adults | 3 (100%) | 0  (0.0%) | 0  (0.0%) | MD1: But needs to be linked to sedation protocols and daily awakening trials. |
| 2 | Sedation protocols (minimize sedation use) | Sedation protocols aimed at minimizing sedation. | 3 (100%) | 0  (0.0%) | 0  (0.0%) | MD1: But protocols may be site specific and does not have to be universally the same for all. Some sites will do protocols with daily awakening trials. Other sites want protocols that titrates to target RASS that is usually 0. Others suggest opioid only protocol and others want bolus only initially and continuous drips only if needed after boluses.  MD2: In some form—it would have to be defined to be measurable, etc. |
| 3 | Cuff leak test in high risk patients | Perform cuff leak test in MV adults who meet extubation criteria and are deemed high risk for post-extubation stridor. | 1 (33.3%) | 0  (0.0%) | 2 (66.6%) | MD1: Easy to do. Controversy is whether you should avoid extubation if there is no cuff leak. Clinicians should decide whether to just extubate, wait and give steroids. But I feel that knowing is still better than not knowing if there is a cuff leak. |
| 3 | Short term steroids for patients who fail cuff leak test | For adults who have failed cuff leak test but are otherwise ready for extubation, administer steroids for at least 4 hours before extubation. | 2 (66.6%) | 0  (0.0%) | 1 (33.3%) | MD1: Should not be dictated but given as an option to consider. |
| 3 | Ventilator liberation protocol | Manage acutely hospitalized adults who have been MV >24 hours with a ventilator liberation protocol (designed to reduce variation in practice). | 3 (100%) | 0  (0.0%) | 0  (0.0%) |  |
| 3 | Extubation to preventive non-invasive ventilation | Extubation to preventive non-invasive ventilation (NIV). | 1 (33.3%) | 1 (33.3%) | 1 (33.3%) | MD1: Only for COPD and patients with hypercapnic respiratory failure.  MD2: As written, it isn’t clear who the population is. Would need to drill down on the population with the greatest potential benefit since most patients don’t need this.  MD3: HHFNC |
| 3 | Extubation to high flow nasal cannula | Extubation to high flow nasal cannula. | 2 (66.6%) | 0  (0.0%) | 1 (33.3%) | MD1: Only for hypoxemic respiratory failure or patients at high risk for failure.  MD2: Same as my response to extubate to NIV. |

**Table: Detailed results of Step 2: Clinician team members (N=3) decisions on inclusion of specific EBPs from Step 1**

*Reported as N number of participants and % of respondents

ARDS=acute respiratory distress syndrome; MV=invasive mechanical ventilation; NMB=neuromuscular blockade; PEEP=positive end-expiratory pressure; SD=standard deviation
