# Supplementary material for: Prioritizing evidence-based practices for acute respiratory distress syndrome using digital data: an iterative multi-stakeholder process
Source: Implement Sci. 2022 Dec 16;17:82. doi: 10.1186/s13012-022-01255-y (PMC9756680; doi:10.1186/s13012-022-01255-y)
Supplement: Supplementary file 3 — Additional file 3. Combined surveys for Steps 2 and 3. [file 13012_2022_1255_MOESM3_ESM.pdf]

DIGITAL-C Researcher EBP Survey

Start of Block: Intro

Intro Welcome to the DIGITAL-C researcher survey for assessment of evidence-based practices (EBPs).

You will be presented with 26 EBPs, and will be asked to assess each one on a number of criteria to help us evaluate whether to include the EBP as a focus of our interventions. These EBPs have been selected to be across the continuum of care for ARDS, including:
Intubation and initiation of mechanical ventilation Prevention of complications of acute critical illness and hospital-acquired adverse events Extubation and discontinuation of mechanical ventilation

---

Name Name

____

Defs Please open up the spreadsheet linked here for reference throughout the survey. Review the definitions of the assessment criteria below before beginning the survey. There will be a chance for you to comment on each EBP, and the EBPs overall.

Measurable- Endpoints or markers are well identified in this EBP to make it easily measurable and computable in the EHR. **Clarity of execution**- EBP is clear on how the recommendation should be executed, with "what" and "how" defined, including step-by-step instructions. **Decidable**- EBP has high clarity as to under what conditions to perform the EBP (e.g. age, gender, clinical findings, laboratory results). **Valid**- Recommendation highly reflects the intent of the developer and the quality of evidence. **Flexible**- The recommendation permits interpretation and allows for alternatives in its execution. **Effect on process of care**- The recommendation can be carried out without substantial disruption of current workflow or significant increased need for resources. **Novel/innovative**- The recommendation proposes behaviors considered new and unconventional by clinicians (or patients). **Resource Intensive**- The EBP is resource intensive. **Clarity of target**

population- The guideline clearly defines the target patient population. **Credible author**- The organizations and authors who developed the guideline have credibility with the intended audience of the guideline. **Consistency**- The recommendation is consistent among other authors in the literature and your understanding of evidence based practice.

End of Block: Intro

Start of Block: 1-Initiation

Ini_Intro The following questions will focus on EBPs about intubation and initiation of mechanical ventilation. Please see below for criteria definitions for your reference.

Ini_Defs
Measurable- Endpoints or markers are well identified in this EBP to make it easily measurable and computable in the EHR. **Clarity of execution**- EBP is clear on how the recommendation should be executed, with "what" and "how" defined, including step-by-step instructions. **Decidable**- EBP has high clarity as to under what conditions to perform the EBP (e.g. age, gender, clinical findings, laboratory results). **Valid**- Recommendation highly reflects the intent of the developer and the quality of evidence. **Flexible**- The recommendation permits interpretation and allows for alternatives in its execution. **Effect on process of care**- The recommendation can be carried out without substantial disruption of current workflow or significant increased need for resources. **Novel/innovative**- The recommendation proposes behaviors considered new and unconventional by clinicians (or patients). **Resource Intensive**- The EBP is resource intensive. **Clarity of target population**- The guideline clearly defines the target patient population. **Credible author**- The organizations and authors who developed the guideline have credibility with the intended audience of the guideline. **Consistency**- The recommendation is consistent among other authors in the literature and your understanding of evidence based practice.

1.1_LPV
EBP 1.1 (Fan et al. 2017)
Lung Protective Ventilation

Recommendation: Mechanical ventilation using lower tidal volumes (4–8 ml/kg predicted body weight) and lower inspiratory pressures (plateau pressure < 30 cm H_2O) for adult patients with ARDS

Please rank the degree to which you agree that criteria below apply to the EBP above.

	1- Highly Disagree (1)	2 (2)	3 (3)	4 (4)	5- Highly Agree (5)
Measurable (1.1_Measure)	○	○	○	○	○
Clarity on execution (1.1_Clarity)	○	○	○	○	○
Decidable (1.1_Decidability)	○	○	○	○	○
Valid (1.1_Validity)	○	○	○	○	○
Flexible (1.1_Flexibility)	○	○	○	○	○
Effect on process of care (1.1_Process)	○	○	○	○	○
Novelty/Innovation (1.1_Novelty)	○	○	○	○	○
Resource Intensiveness (1.1_Resource)	○	○	○	○	○
Clarity on target population (1.1_Pop)	○	○	○	○	○
Author credibility (1.1_Cred)	○	○	○	○	○
Consistency (1.1_Consistent)	○	○	○	○	○

1.1_LPV_Text Please comment on any additional thoughts about the above EBP below:

____

____

1.4_PEEP
EBP 1.4 (Fan et al. 2017)
High PEEP

Recommendation: Higher positive end-expiratory pressure in patients (PEEP) with moderate or severe ARDS

Please rank the degree to which you agree that criteria below apply to the EBP above.

	1- Highly Disagree (1)	2 (2)	3 (3)	4 (4)	5- Highly Agree (5)
Measurable (1.4_Measure)	○	○	○	○	○
Clarity on execution (1.4_Clarity)	○	○	○	○	○
Decidable (1.4_Decidability)	○	○	○	○	○
Valid (1.4_Validity)	○	○	○	○	○
Flexible (1.4_Flexibility)	○	○	○	○	○
Effect on process of care (1.4_Process)	○	○	○	○	○
Novelty/Innovation (1.4_Novelty)	○	○	○	○	○
Resource Intensiveness (1.4_Resource)	○	○	○	○	○
Clarity on target population (1.4_Pop)	○	○	○	○	○
Author credibility (1.4_Cred)	○	○	○	○	○
Consistency (1.4_Consistent)	○	○	○	○	○

1.4_PEEP_Text Please comment on any additional thoughts about the above EBP below:

____

____

1.5_Recruit
EBP 1.5 (Fan et al. 2017)
Recruitment maneuvers

Recommendation: Recruitment maneuvers for adult patients with moderate or severe ARDS

Please rank the degree to which you agree that criteria below apply to the EBP above.

	1- Highly Disagree (1)	2 (2)	3 (3)	4 (4)	5- Highly Agree (5)
Measurable (1.5_Measure)	○	○	○	○	○
Clarity on execution (1.5_Clarity)	○	○	○	○	○
Decidable (1.5_Decidability)	○	○	○	○	○
Valid (1.5_Validity)	○	○	○	○	○
Flexible (1.5_Flexibility)	○	○	○	○	○
Effect on process of care (1.5_Process)	○	○	○	○	○
Novelty/Innovation (1.5_Novelty)	○	○	○	○	○
Resource Intensiveness (1.5_Resource)	○	○	○	○	○
Clarity on target population (1.5_Pop)	○	○	○	○	○
Author credibility (1.5_Cred)	○	○	○	○	○
Consistency (1.5_Consistent)	○	○	○	○	○

1.5_Recruit_Text Please comment on any additional thoughts about the above EBP below:

____

____

1.3_HFOV
EBP 1.3 (Fan et al. 2017)
[Merged with EBP 3.7 12.1 (Goligher 2017)]
HFOV

Recommendation: Do not use high-frequency oscillatory ventilation (HFOV) in patients with moderate or severe ARDS. HFOV is not associated with a mortality benefit, and may even be harmful in comparison to ventilation with low tidal volumes and higher levels of PEEP.

Please rank the degree to which you agree that criteria below apply to the EBP above.

	1-Highly Disagree (1)	2 (2)	3 (3)	4 (4)	5- Highly agree (5)
Measurable (1.3_Measure)	○	○	○	○	○
Clarity on execution (1.3_Clarity)	○	○	○	○	○
Decidable (1.3_Decidability)	○	○	○	○	○
Valid (1.3_Validity)	○	○	○	○	○
Flexibile (1.3_Flexibility)	○	○	○	○	○
Effect on process of care (1.3_Process)	○	○	○	○	○
Novelty/Innovation (1.3_Novelty)	○	○	○	○	○
Resource Intensiveness (1.3_Resource)	○	○	○	○	○
Clarity on target population (1.3_Clarity)	○	○	○	○	○
Author credibility (1.3_Cred)	○	○	○	○	○
Consistency (1.3_Consistent)	○	○	○	○	○

1.3_HFOV_Text Please comment on any additional thoughts about the above EBP below:

____

____

15.1_NMB
EBP 15.1 (Annane 2016)
Neuromuscular blockade

Recommendation: For rapid sequence intubation, a single intravenous bolus of a short-acting curare such as succinylcholine is recommended to permit transient full relaxation of the jaw and larynx muscles, facilitating the introduction of the tracheal tube.

Please rank the degree to which you agree that criteria below apply to the EBP above.

	1-Highly Disagree (1)	2 (2)	3 (3)	4 (4)	5-Highly Agree (5)
Measurable (15.1_Measure)	○	○	○	○	○
Clarity on execution (15.1_Clarity)	○	○	○	○	○
Decidable (15.1_Decidability)	○	○	○	○	○
Valid (15.1_Validity)	○	○	○	○	○
Flexible (15.1_Flexibility)	○	○	○	○	○
Effect on process of care (15.1_Process)	○	○	○	○	○
Novelty/Innovation (15.1_Novelty)	○	○	○	○	○
Resource Intensiveness (15.1_Resource)	○	○	○	○	○
Clarity on target population (15.1_Pop)	○	○	○	○	○
Author credibility (15.1_Cred)	○	○	○	○	○
Consistency (15.1_Consistent)	○	○	○	○	○

15.1_NMB_Text Please comment on any additional thoughts about the above EBP below:

____

____

____

____

____

End of Block: 1-Initiation

Start of Block: 2-Prevention

Prevent_Intro The following questions will focus on EBPs about prevention of complications of acute critical illness and hospital acquired adverse events. Please see below for criteria definitions for your reference.

Prevent_Defs
Measurable- Endpoints or markers are well identified in this EBP to make it easily measurable and computable in the EHR. **Clarity of execution**- EBP is clear on how the recommendation should be executed, with "what" and "how" defined, including step-by-step instructions. **Decidable**- EBP has high clarity as to under what conditions to perform the EBP (e.g. age, gender, clinical findings, laboratory results). **Valid**- Recommendation highly reflects the intent of the developer and the quality of evidence. **Flexible**- The recommendation permits interpretation and allows for alternatives in its execution. **Effect on process of care**- The recommendation can be carried out without substantial disruption of current workflow or significant increased need for resources. **Novel/innovative**- The recommendation proposes behaviors considered new and unconventional by clinicians (or patients). **Resource Intensive**- The EBP is resource intensive. **Clarity of target population**- The guideline clearly defines the target patient population. **Credible author**- The organizations and authors who developed the guideline have credibility with the intended audience of the guideline. **Consistency**- The recommendation is consistent among other authors in the literature and your understanding of evidence based practice.

X→ X→

4.25_Sedat
EBP 4.25 (Devlin et al. 2018)
Sedation protocols

Recommendation: Using either propofol or dexmedetomidine is preffered over benzodiazepines for sedation in critically ill, mechanically ventilated adults

Please rank the degree to which you agree that criteria below apply to the EBP above.

	1-Highly Disagree (1)	2 (2)	3 (3)	4 (4)	5- Highly Agree (5)
Measurable (4.25_Measure)	○	○	○	○	○
Clarity on execution (4.25_Clarity)	○	○	○	○	○
Decidable (4.25_Decidability)	○	○	○	○	○
Valid (4.25_Validity)	○	○	○	○	○
Flexible (4.25_Flexibility)	○	○	○	○	○
Effect on process of care (4.25_Process)	○	○	○	○	○
Novelty/Innovation (4.25_Novelty)	○	○	○	○	○
Resource Intensiveness (4.25_Resource)	○	○	○	○	○
Clarity on target population (4.25_Pop)	○	○	○	○	○
Author credibility (4.25_Cred)	○	○	○	○	○
Consistency (4.25_Consistent)	○	○	○	○	○

4.25_Sedat_Text Please comment on any additional thoughts about the above EBP below:

____

____

6.2_Sedat
EBP 6.2 (Ouellette et al. 2017)
Sedation protocols

Recommendation: Sedation protocols aimed at minimizing sedation.

Please rank the degree to which you agree that criteria below apply to the EBP above.

	1-Highly Disagree (1)	2 (2)	3 (3)	4 (4)	5-Highly Agree (5)
Measurable (6.2_Measure)	○	○	○	○	○
Clarity on execution (6.2_Clarity)	○	○	○	○	○
Decidable (6.2_Decidability)	○	○	○	○	○
Valid (6.2_Validity)	○	○	○	○	○
Flexible (6.2_Flexibility)	○	○	○	○	○
Effect on process of care (6.2_Process)	○	○	○	○	○
Novelty/Innovation (6.2_Novelty)	○	○	○	○	○
Resource Intensiveness (6.2_Resource)	○	○	○	○	○
Clarity on target population (6.2_Pop)	○	○	○	○	○
Author credibility (6.2_Cred)	○	○	○	○	○
Consistency (6.2_Consistent)	○	○	○	○	○

6.2_Sedat_Text Please comment on any additional thoughts about the above EBP below:

____

____

1.2_Prone
EBP 1.2 (Fan et al. 2017)
Prone Positioning

Recommendation: Prone positioning for more than 12 hours/day for adult patients with severe ARDS.

Please rank the degree to which you agree that criteria below apply to the EBP above.

	1- Highly Disagree (1)	2 (2)	3 (3)	4 (4)	5- Highly Agree (5)
Measurable (1.2_Measure)	○	○	○	○	○
Clarity on execution (1.2_Clarity)	○	○	○	○	○
Decidable (1.2_Decidability)	○	○	○	○	○
Valid (1.2_Validity)	○	○	○	○	○
Flexible (1.2_Flexibility)	○	○	○	○	○
Effect on process of care (1.2_Process)	○	○	○	○	○
Novelty/Innovation (1.2_Novelty)	○	○	○	○	○
Resource Intensiveness (1.2_Resource)	○	○	○	○	○
Clarity on target population (1.2_Pop)	○	○	○	○	○
Author credibility (1.2_Cred)	○	○	○	○	○
Consistency (1.2_Consistent)	○	○	○	○	○

1.2_Prone_Text Please comment on any additional thoughts about the above EBP below:

____

____

4.23_Daily
EBP 4.23 (Devlin et al. 2018)
Daily awakening and breathing trials

Recommendation: Use of daily sedative interruption (DSI)/nurse-protocolized sedation to achieve and maintain a light level of sedation

Please rank the degree to which you agree that criteria below apply to the EBP above.

	1-Highly Disagree (1)	2 (2)	3 (3)	4 (4)	5- Highly Agree (5)
Measurable (4.23_Measure)	○	○	○	○	○
Clarity on execution (4.23_Clarity)	○	○	○	○	○
Decidable (4.23_Decidability)	○	○	○	○	○
Valid (4.23_Validity)	○	○	○	○	○
Flexible (4.23_Flexibility)	○	○	○	○	○
Effect on process of care (4.23_Process)	○	○	○	○	○
Novelty/Innovation (4.23_Novelty)	○	○	○	○	○
Resource Intensiveness (4.23_Resource)	○	○	○	○	○
Clarity on target population (4.23_Pop)	○	○	○	○	○
Author credibility (4.23_Cred)	○	○	○	○	○
Consistency (4.23_Consistent)	○	○	○	○	○

4.23_Daily_Text Please comment on any additional thoughts about the above EBP below:

____

____

4.2_Pain
EBP 4.21 (Devlin et al. 2018)
Protocol-based pain assessment and management

Recommendation: Routine use of an assessment-driven, protocol-based, stepwise approach for pain and sedation management in critically ill adults. Pain should be treated before a sedative agent is considered.

Please rank the degree to which you agree that criteria below apply to the EBP above.

	1-Highly Disagree (1)	2 (2)	3 (3)	4 (4)	5-Highly Agree (5)
Measurable (4.2_Measure)	○	○	○	○	○
Clarity on execution (4.2_Clarity)	○	○	○	○	○
Decidable (4.2_Decidability)	○	○	○	○	○
Valid (4.2_Validity)	○	○	○	○	○
Flexible (4.2_Flexibility)	○	○	○	○	○
Effect on process of care (4.2_Process)	○	○	○	○	○
Novelty/Innovation (4.2_Novelty)	○	○	○	○	○
Resource Intensiveness (4.2_Resource)	○	○	○	○	○
Clarity on target population (4.2_Pop)	○	○	○	○	○
Author credibility (4.2_Cred)	○	○	○	○	○
Consistency (4.2_Consistent)	○	○	○	○	○

4.2_Pain_Text Please comment on any additional thoughts about the above EBP below:

____

____

6.1_Pain
EBP 6.1 (Ouellette et al. 2017)
[Merged with EBP 3.2 (Schmidt et al. 2016)]
Protocol-based pain assessment and management

Recommendation: SBT conducted with inspiratory pressure augmentation (5-8 cm H2O, with pressure support ventilation, automatic tube compensation) rather than without (T-piece or CPAP).

Please rank the degree to which you agree that criteria below apply to the EBP above.

	1-Highly Disagree (1)	2 (2)	3 (3)	4 (4)	5-Highly Agree (5)
Measurable (6.1_Measure)	○	○	○	○	○
Clarity on execution (6.1_Clarity)	○	○	○	○	○
Decidable (6.1_Decidability)	○	○	○	○	○
Valid (6.1_Validity)	○	○	○	○	○
Flexible (6.1_Flexibility)	○	○	○	○	○
Effect on process of care (6.1_Process)	○	○	○	○	○
Novelty/Innovation (6.1_Novelty)	○	○	○	○	○
Resource Intensiveness (6.1_Resource)	○	○	○	○	○
Clarity on target population (6.1_Pop)	○	○	○	○	○
Author credibility (6.1_Cred)	○	○	○	○	○
Consistency (6.1_Consistent)	○	○	○	○	○

6.1_Pain_Text Please comment on any additional thoughts about the above EBP below:

____

____

4.30_Deler
EBP 4.30 (Devlin et al. 2018)
Delerium assessment/prevention

Recommendation: Regular assessment of delirium using validated screening tools for critically ill adults.

Please rank the degree to which you agree that criteria below apply to the EBP above.

	1-Highly Disagree (1)	2 (2)	3 (3)	4 (4)	5-Highly Agree (5)
Measurable (4.30_Measure)	○	○	○	○	○
Clarity on execution (4.30_Clarity)	○	○	○	○	○
Decidable (4.30_Decidability)	○	○	○	○	○
Valid (4.30_Validity)	○	○	○	○	○
Flexible (4.30_Flexibility)	○	○	○	○	○
Effect on process of care (4.30_Process)	○	○	○	○	○
Novelty/Innovation (4.30_Novelty)	○	○	○	○	○
Resource Intensiveness (4.30_Resource)	○	○	○	○	○
Clarity on target population (4.30_Pop)	○	○	○	○	○
Author credibility (4.30_Cred)	○	○	○	○	○
Consistency (4.30_Consistent)	○	○	○	○	○

4.30_Deler_Text Please comment on any additional thoughts about the above EBP below:

____

____

4.51_Deler
EBP 4.3 (Devlin et al. 2018)
Delerium assessment/prevention

Recommendation: Using noise and light reduction strategies to improve sleep in critically ill adults.

Please rank the degree to which you agree that criteria below apply to the EBP above.

	1-Highly Disagree (1)	2 (2)	3 (3)	4 (4)	5-Highly Agree (5)
Measurable (4.51_Measure)	○	○	○	○	○
Clarity on execution (4.51_Clarity)	○	○	○	○	○
Decidable (4.51_Decidability)	○	○	○	○	○
Valid (4.51_Validity)	○	○	○	○	○
Flexible (4.51_Flexibility)	○	○	○	○	○
Effect on process of care (4.51_Process)	○	○	○	○	○
Novelty/Innovation (4.51_Novelty)	○	○	○	○	○
Resource Intensiveness (4.51_Resource)	○	○	○	○	○
Clarity on target population (4.51_Pop)	○	○	○	○	○
Author credibility (4.51_Cred)	○	○	○	○	○
Consistency (4.51_Consistent)	○	○	○	○	○

4.51_Deler_Text Please comment on any additional thoughts about the above EBP below:

____

____

4.42_Mobil
EBP 4.42 (Devlin et al. 2018)
[Merged with EBP 14.1 (Dammeyer et al. 2013)]
Early mobilization

Recommendation: Performing rehabilitation or mobilization in critically ill adults with mobility protocols executed by nurses and PTs with family involvement.

Please rank the degree to which you agree that criteria below apply to the EBP above.

	1-Highly Disagree (1)	2 (2)	3 (3)	4 (4)	5- Highly Agree (5)
Measurable (4.42_Measure)	○	○	○	○	○
Clarity on execution (4.42_Clarity)	○	○	○	○	○
Decidable (4.42_Decidability)	○	○	○	○	○
Valid (4.42_Validity)	○	○	○	○	○
Flexible (4.42_Flexibility)	○	○	○	○	○
Effect on process of care (4.42_Process)	○	○	○	○	○
Novelty/Innovation (4.42_Novelty)	○	○	○	○	○
Resource Intensiveness (4.42_Resource)	○	○	○	○	○
Clarity on target population (4.42_Pop)	○	○	○	○	○
Author credibility (4.42_Cred)	○	○	○	○	○
Consistency (4.42_Consistent)	○	○	○	○	○

4.42_Mobil_Text Please comment on any additional thoughts about the above EBP below:

____

____

15.2_NMB
EBP 15.2 (Murray 2016)
Neuromuscular blockade

Recommendation: Scheduled eye care that includes lubricating drops or gel and eyelid closure for patients receiving continuous neuromuscular-blockading agents.

Please rank the degree to which you agree that criteria below apply to the EBP above.

	1-Highly Disagree (1)	2 (2)	3 (3)	4 (4)	5- Highly Agree (5)
Measurable (15.2_Measure)	◯	◯	◯	◯	◯
Clarity on execution (15.2_Clarity)	◯	◯	◯	◯	◯
Decidable (15.2_Decidability)	◯	◯	◯	◯	◯
Valid (15.2_Validity)	◯	◯	◯	◯	◯
Flexible (15.2_Flexibility)	◯	◯	◯	◯	◯
Effect on process of care (15.2_Process)	◯	◯	◯	◯	◯
Novelty/Innovation (15.2_Novelty)	◯	◯	◯	◯	◯
Resource Intensiveness (15.2_Resource)	◯	◯	◯	◯	◯
Clarity on target population (15.2_Pop)	◯	◯	◯	◯	◯
Author credibility (15.2_Cred)	◯	◯	◯	◯	◯
Consistency (15.2_Consistent)	◯	◯	◯	◯	◯

15.2_NMB_Text Please comment on any additional thoughts about the above EBP below:

____

____

15.3_NMB
EBP 15.3 (Murray 2016)
Neuromuscular blockade

Recommendation: Neuromuscular-blocking agent administered by continuous intravenous infusion early in the course of acute respiratory distress syndrome for patients with a Pao2/Fio2 less than 150.

Please rank the degree to which you agree that criteria below apply to the EBP above.

	1-Highly Disagree (1)	2 (2)	3 (3)	4 (4)	5- Highly Agree (5)
Measurable (15.3_Measure)	○	○	○	○	○
Clarity on execution (15.3_Clarity)	○	○	○	○	○
Decidable (15.3_Decidability)	○	○	○	○	○
Valid (15.3_Validity)	○	○	○	○	○
Flexible (15.3_Flexibility)	○	○	○	○	○
Effect on process of care (15.3_Process)	○	○	○	○	○
Novelty/Innovation (15.3_Novelty)	○	○	○	○	○
Resource Intensiveness (15.3_Resource)	○	○	○	○	○
Clarity on target population (15.3_Pop)	○	○	○	○	○
Author credibility (15.3_Cred)	○	○	○	○	○
Consistency (15.3_Consistent)	○	○	○	○	○

15.3_NMB_Text Please comment on any additional thoughts about the above EBP below:

____

____

15.5_NMB
EBP 15.5 (Murray 2016)
Neuromuscular blockade

Recommendation: Use/trial of a neuromuscular-blocking agents in life-threatening situations associated with profound hypoxemia, respiratory acidosis, or hemodynamic compromise.

Please rank the degree to which you agree that criteria below apply to the EBP above.

	1- Highly Disagree (1)	2 (2)	3 (3)	4 (4)	5- Highly Agree (5)
Measurable (15.5_Measure)	○	○	○	○	○
Clarity on execution (15.5_Clarity)	○	○	○	○	○
Decidable (15.5_Decidability)	○	○	○	○	○
Valid (15.5_Validity)	○	○	○	○	○
Flexible (15.5_Flexibility)	○	○	○	○	○
Effect on process of care (15.5_Process)	○	○	○	○	○
Novelty/Innovation (15.5_Novelty)	○	○	○	○	○
Resource Intensiveness (15.5_Resource)	○	○	○	○	○
Clarity on target population (15.5_Pop)	○	○	○	○	○
Author credibility (15.5_Cred)	○	○	○	○	○
Consistency (15.5_Consistent)	○	○	○	○	○

15.5_NMB_Text Please comment on any additional thoughts about the above EBP below:

____

____

15.9_NMB
EBP 15.9 (Murray 2016)
Neuromuscular blockade

Recommendation: Patients receiving a continuous infusion of neuromuscular-blocking agent should receive a structured physiotherapy regimen.

Please rank the degree to which you agree that criteria below apply to the EBP above.

	1- Highly Disagree (1)	2 (2)	3 (3)	4 (4)	5- Highly Agree (5)
Measurable (15.9_Measure)	○	○	○	○	○
Clarity on execution (15.9_Clarity)	○	○	○	○	○
Decidable (15.9_Decidability)	○	○	○	○	○
Valid (15.9_Validity)	○	○	○	○	○
Flexible (15.9_Flexibility)	○	○	○	○	○
Effect on process of care (15.9_Process)	○	○	○	○	○
Novelty/Innovation (15.9_Novelty)	○	○	○	○	○
Resource Intensiveness (15.9_Resource)	○	○	○	○	○
Clarity on target population (15.9_Pop)	○	○	○	○	○
Author credibility (15.9_Cred)	○	○	○	○	○
Consistency (15.9_Consistent)	○	○	○	○	○

15.9_NMB_Text Please comment on any additional thoughts about the above EBP below:

____

____

15.10_NMB
EBP 15.10 (Murray 2016)
Neuromuscular blockade

Recommendation: Target blood glucose level for less than 180mg/dL in patients receiving neuromuscular-blocking agents.

Please rank the degree to which you agree that criteria below apply to the EBP above.

	1- Highly Disagree (1)	2 (2)	3 (3)	4 (4)	5- Highly Agree (5)
Measurable (15.10_Measure)	○	○	○	○	○
Clarity on execution (15.10_Clarity)	○	○	○	○	○
Decidable (15.10_Decidability)	○	○	○	○	○
Valid (15.10_Validity)	○	○	○	○	○
Flexible (15.10_Flexibility)	○	○	○	○	○
Effect on process of care (15.10_Process)	○	○	○	○	○
Novelty/Innovation (15.10_Novelty)	○	○	○	○	○
Resource Intensiveness (15.10_Resource)	○	○	○	○	○
Clarity on target population (15.10_Pop)	○	○	○	○	○
Author credibility (15.10_Cred)	○	○	○	○	○
Consistency (15.10_Consistent)	○	○	○	○	○

15.10_NMB Please comment on any additional thoughts about the above EBP below:

____

____

2.1_LOT
EBP 2.1 (Chu et al. 2018)
Liberal oxygen therapy

Recommendation: Recommend against liberal oxygen therapy in acutely ill adults (aged ≥18 years) defined as saturation of peripheral oxygen [SpO_2] above range of 94% to 96%.

Please rank the degree to which you agree that criteria below apply to the EBP above.

	1- Highly Disagree (1)	2 (2)	3 (3)	4 (4)	5- Highly Agree (5)
Measurable (2.1_Measure)	○	○	○	○	○
Clarity on execution (2.1_Clarity)	○	○	○	○	○
Decidable (2.1_Decidability)	○	○	○	○	○
Valid (2.1_Validity)	○	○	○	○	○
Flexible (2.1_Flexibility)	○	○	○	○	○
Effect on process of care (2.1_Process)	○	○	○	○	○
Novelty/Innovation (2.1_Novelty)	○	○	○	○	○
Resource Intensiveness (2.1_Resource)	○	○	○	○	○
Clarity on target population (2.1_Pop)	○	○	○	○	○
Author credibility (2.1_Cred)	○	○	○	○	○
Consistency (2.1_Consistent)	○	○	○	○	○

2.1_LOT_Text Please comment on any additional thoughts about the above EBP below:

____

____

18.1_OLS
EBP 18.1 (Lu 2017)
Open lung strategy

Recommendation: OLS (open lung strategy) during mechanical ventilation for patients with ARDS.

Please rank the degree to which you agree that criteria below apply to the EBP above.

	1-Highly Disagree (1)	2 (2)	3 (3)	4 (4)	5- Highly Agree (5)
Measurable (18.1_Measure)	○	○	○	○	○
Clarity on execution (18.1_Clarity)	○	○	○	○	○
Decidable (18.1_Decidability)	○	○	○	○	○
Valid (18.1_Validity)	○	○	○	○	○
Flexible (18.1_Flexibility)	○	○	○	○	○
Effect on process of care (18.1_Process)	○	○	○	○	○
Novelty/Innovation (18.1_Novelty)	○	○	○	○	○
Resource Intensiveness (18.1_Resource)	○	○	○	○	○
Clarity on target population (18.1_Pop)	○	○	○	○	○
Author credibility (18.1_Cred)	○	○	○	○	○
Consistency (18.1_Consistent)	○	○	○	○	○

18.1_OLS_Text Please comment on any additional thoughts about the above EBP below:

____

____

____

____

____

End of Block: 2-Prevention

Start of Block: 3-Discontinuation

Dis_Intro The following questions will focus on EBPs about extubation and discontinuation of mechanical ventilation. Please see below for criteria definitions for your reference.

Dis_Defs
Measurable- Endpoints or markers are well identified in this EBP to make it easily measurable and computable in the EHR. **Clarity of execution**- EBP is clear on how the recommendation should be executed, with "what" and "how" defined, including step-by-step instructions. **Decidable**- EBP has high clarity as to under what conditions to perform the EBP (e.g. age, gender, clinical findings, laboratory results). **Valid**- Recommendation highly reflects the intent of the developer and the quality of evidence. **Flexible**- The recommendation permits interpretation and allows for alternatives in its execution. **Effect on process of care**- The recommendation can be carried out without substantial disruption of current workflow or significant increased need for resources. **Novel/innovative**- The recommendation proposes behaviors considered new and unconventional by clinicians (or patients). **Resource Intensive**- The EBP is resource intensive. **Clarity of target population**- The guideline clearly defines the target patient population. **Credible author**- The organizations and authors who developed the guideline have credibility with the intended audience of the guideline. **Consistency**- The recommendation is consistent among other authors in the literature and your understanding of evidence based practice.

X→ X→

5.3_Cuff
EBP 5.3 (Girard et al. 2016)
Cuff leak test in high risk patients

Recommendation: Perform cuff leak test in MV adults who meet extubation criteria and are deemed high risk for post-extubation stridor

Please rank the degree to which you agree that criteria below apply to the EBP above.

	1- Highly Disagree (1)	2 (2)	3 (3)	4 (4)	5- Highly Agree (5)
Measurable (5.3_Measure)	○	○	○	○	○
Clarity on execution (5.3_Clarity)	○	○	○	○	○
Decidable (5.3_Decidability)	○	○	○	○	○
Valid (5.3_Validity)	○	○	○	○	○
Flexible (5.3_Flexibility)	○	○	○	○	○
Effect on process of care (5.3_Process)	○	○	○	○	○
Novelty/Innovation (5.3_Novelty)	○	○	○	○	○
Resource Intensiveness (5.3_Resource)	○	○	○	○	○
Clarity on target population (5.3_Pop)	○	○	○	○	○
Author credibility (5.3_Cred)	○	○	○	○	○
Consistency (5.3_Consistent)	○	○	○	○	○

5.3_Cuff_Text Please comment on any additional thoughts about the above EBP below:

____

____

5.1_Mobil
EBP 5.1 (Girard et al. 2016)
Early mobilization

Recommendation: For acutely hospitalized patients who have ben mechanically ventilated for >24h, suggest protocolized rehabilitation directed toward early mobilization.

Please rank the degree to which you agree that criteria below apply to the EBP above.

	1- Highly Disagree (1)	2 (2)	3 (3)	4 (4)	5- Highly Agree (5)
Measurable (5.1_Measure)	○	○	○	○	○
Clarity on execution (5.1_Clarity)	○	○	○	○	○
Decidable (5.1_Decidability)	○	○	○	○	○
Valid (5.1_Validity)	○	○	○	○	○
Flexible (5.1_Flexibility)	○	○	○	○	○
Effect on process of care (5.1_Process)	○	○	○	○	○
Novelty/Innovation (5.1_Novelty)	○	○	○	○	○
Resource Intensiveness (5.1_Resource)	○	○	○	○	○
Clarity on target population (5.1_Pop)	○	○	○	○	○
Author credibility (5.1_Cred)	○	○	○	○	○
Consistency (5.1_Consistent)	○	○	○	○	○

5.1_Mobil_Text Please comment on any additional thoughts about the above EBP below:

____

____

5.2_VentPro
EBP 5.2 (Girard et al. 2016)
Ventilator liberation protocol

Recommendation: Manage acutely hosp adults who have been MV >24 hours with a ventilator liberation protocol (designed to reduce variation in practice).

Please rank the degree to which you agree that criteria below apply to the EBP above.

	1- Highly Disagree (1)	2 (2)	3 (3)	4 (4)	5- Highly Agree (5)
Measurable (5.2_Measure)	o	o	o	o	o
Clarity on execution (5.2_Clarity)	o	o	o	o	o
Decidable (5.2_Decidability)	o	o	o	o	o
Valid (5.2_Validity)	o	o	o	o	o
Flexible (5.2_Flexibility)	o	o	o	o	o
Effect on process of care (5.2_Process)	o	o	o	o	o
Novelty/Innovation (5.2_Novelty)	o	o	o	o	o
Resource Intensiveness (5.2_Resource)	o	o	o	o	o
Clarity on target population (5.2_Pop)	o	o	o	o	o
Author credibility (5.2_Cred)	o	o	o	o	o
Consistency (5.2_Consistent)	o	o	o	o	o

5.2_VentPro_Text Please comment on any additional thoughts about the above EBP below:

____

____

5.4_Steroid
EBP 5.4 (Girard et al. 2016)
[Merged with EBP 3.7 (Schmidt et al. 2016)]
Short term steroids for high risk patients who fail cuff leak test

Recommendation: For adults who have failed cuff leak test but are otherwise ready for extubation, administer systemic steroids for at least 4 hours before extubation

Please rank the degree to which you agree that criteria below apply to the EBP above.

	1- Highly Disagree (1)	2 (2)	3 (3)	4 (4)	5- Highly Agree (5)
Measurable (5.4_Measure)	○	○	○	○	○
Clarity on execution (5.4_Clarity)	○	○	○	○	○
Decidable (5.4_Decidability)	○	○	○	○	○
Valid (5.4_Validity)	○	○	○	○	○
Flexible (5.4_Flexibility)	○	○	○	○	○
Effect on process of care (5.4_Process)	○	○	○	○	○
Novelty/Innovation (5.4_Novelty)	○	○	○	○	○
Resource Intensiveness (5.4_Resource)	○	○	○	○	○
Clarity on target population (5.4_Pop)	○	○	○	○	○
Author credibility (5.4_Cred)	○	○	○	○	○
Consistency (5.4_Consistent)	○	○	○	○	○

5.4_Steroid_Text Please comment on any additional thoughts about the above EBP below:

____

____

6.3_Ex
EBP 6.3 (Ouellette et al. 2017)
Extubation

Recommendation: Extubation to preventive non-invasive ventilation (NIV).

Please rank the degree to which you agree that criteria below apply to the EBP above.

	1- Highly Disagree (1)	2 (2)	3 (3)	4 (4)	5- Highly Agree (5)
Measurable (6.3_Measure)	○	○	○	○	○
Clarity on execution (6.3_Clarity)	○	○	○	○	○
Decidable (6.3_Decidability)	○	○	○	○	○
Valid (6.3_Validity)	○	○	○	○	○
Flexible (6.3_Flexibility)	○	○	○	○	○
Effect on process of care (6.3_Process)	○	○	○	○	○
Novelty/Innovation (6.3_Novelty)	○	○	○	○	○
Resource Intensiveness (6.3_Resource)	○	○	○	○	○
Clarity on target population (6.3_Pop)	○	○	○	○	○
Author credibility (6.3_Cred)	○	○	○	○	○
Consistency (6.3_Consistent)	○	○	○	○	○

6.3_Ex_Text Please comment on any additional thoughts about the above EBP below:

____

____

End of Block: 3-Discontinuation

Start of Block: Comments

Q72 Please indicate if you believe there are EBPS that were not included that should have been:

Final_Text Please leave any comments or thoughts you have overall, below:

End of Block: Comments

DIGITAL-C Researcher EBP Importance Survey

Start of Block: Default Question Block

Intro This will be a quick cut survey of importance of each evidence-based practice (EBP) that we are considering for inclusion in DIGITAL-C. These EBPs have been selected to be across the continuum of care for ARDS, including: Intubation and initiation of mechanical ventilation Prevention of complications of acute critical illness and hospital-acquired adverse events Extubation and discontinuation of mechanical ventilation

If you think an EBP should only be considered in concert with another one, please indicate in the comments.

Name Name

____

X→ X→

1.1
EBP 1.1 (Fan et al. 2017)
Lung Protective Ventilation

Recommendation: Mechanical ventilation using lower tidal volumes (4–8 ml/kg predicted body weight) and lower inspiratory pressures (plateau pressure < 30 cm H_2O) for adult patients with ARDS

Based on your expertise as a clinician, do you think this EBP is sufficiently important to include?

- ○ Yes (1)
- ○ Maybe (2)
- ○ No (3)

1.1_Text Please comment on any additional thoughts about the above EBP below:

____

____

____

____

____

1.4 EBP 1.4 (Fan et al. 2017)
High PEEP

Recommendation: Higher positive end-expiratory pressure in patients (PEEP) with moderate or severe ARDS

Based on your expertise as a clinician, do you think this EBP is sufficiently important to include?

- ○ Yes (1)
- ○ Maybe (2)
- ○ No (3)

1.4_Text Please comment on any additional thoughts about the above EBP below:

____

____

____

____

____

1.5
EBP 1.5 (Fan et al. 2017)
Recruitment maneuvers

Recommendation: Recruitment maneuvers for adult patients with moderate or severe ARDS

Based on your expertise as a clinician, do you think this EBP is sufficiently important to include?

- Yes (1)
- Maybe (2)
- No (3)

1.5_Text Please comment on any additional thoughts about the above EBP below:

____

____

____

____

____

1.3
EBP 1.3 (Fan et al. 2017)
[Merged with EBP 3.7 12.1 (Goligher 2017)]
HFOV

Recommendation: Do not use high-frequency oscillatory ventilation (HFOV) in patients with moderate or severe ARDS. HFOV is not associated with a mortality benefit, and may even be harmful in comparison to ventilation with low tidal volumes and higher levels of PEEP.

Based on your expertise as a clinician, do you think this EBP is sufficiently important to include?

- ○ Yes (1)
- ○ Maybe (2)
- ○ No (3)

1.3_Text Please comment on any additional thoughts about the above EBP below:

____

____

____

____

____

Page Break

15.1
EBP 15.1 (Annane 2016)
Neuromuscular blockade

Recommendation: For rapid sequence intubation, a single intravenous bolus of a short-acting curare such as succinylcholine is recommended to permit transient full relaxation of the jaw and larynx muscles, facilitating the introduction of the tracheal tube.

Based on your expertise as a clinician, do you think this EBP is sufficiently important to include?

- Yes (1)
- Maybe (2)
- No (3)

15.1_Text Please comment on any additional thoughts about the above EBP below:

____

____

____

____

____

4.25
EBP 4.25 (Devlin et al. 2018)
Sedation protocols

Recommendation: Using either propofol or dexmedetomidine is preffered over benzodiazepines for sedation in critically ill, mechanically ventilated adults

Based on your expertise as a clinician, do you think this EBP is sufficiently important to include?

○ Yes (1)

○ Maybe (2)

○ No (3)

4.25_Text Please comment on any additional thoughts about the above EBP below:

____

____

____

____

____

6.2
EBP 6.2 (Ouellette et al. 2017)
Sedation protocols

Recommendation: Sedation protocols aimed at minimizing sedation.

Based on your expertise as a clinician, do you think this EBP is sufficiently important to include?

○ Yes (1)

○ Maybe (2)

○ No (3)

6.2_Text Please comment on any additional thoughts about the above EBP below:

____

____

____

____

____

1.2
EBP 1.2 (Fan et al. 2017)
Prone Positioning

Recommendation: Prone positioning for more than 12 hours/day for adult patients with severe ARDS.

Based on your expertise as a clinician, do you think this EBP is sufficiently important to include?

- Yes (1)
- Maybe (2)
- No (3)

1.2_Text Please comment on any additional thoughts about the above EBP below:

____

____

____

____

____

4.23
EBP 4.23 (Devlin et al. 2018)
Daily awakening and breathing trials

Recommendation: Use of daily sedative interruption (DSI)/nurse-protocolized sedation to achieve and maintain a light level of sedation

Based on your expertise as a clinician, do you think this EBP is sufficiently important to include?

- ○ Yes (1)
- ○ Maybe (2)
- ○ No (3)

4.23_Text Please comment on any additional thoughts about the above EBP below:

____

____

____

____

____

4.21
EBP 4.21 (Devlin et al. 2018)
Protocol-based pain assessment and management

Recommendation: Routine use of an assessment-driven, protocol-based, stepwise approach for pain and sedation management in critically ill adults. Pain should be treated before a sedative agent is considered.

Based on your expertise as a clinician, do you think this EBP is sufficiently important to include?

○ Yes (1)

○ Maybe (2)

○ No (3)

4.21_Text Please comment on any additional thoughts about the above EBP below:

____

____

____

____

____

6.1
EBP 6.1 (Ouellette et al. 2017)
[Merged with EBP 3.2 (Schmidt et al. 2016)]
Protocol-based pain assessment and management

Recommendation: SBT conducted with inspiratory pressure augmentation (5-8 cm H2O, with pressure support ventilation, automatic tube compensation) rather than without (T-piece or CPAP).

Based on your expertise as a clinician, do you think this EBP is sufficiently important to include?

○ Yes (1)

○ Maybe (2)

○ No (3)

6.1_Text Please comment on any additional thoughts about the above EBP below:

____

____

____

____

____

4.30
EBP 4.30 (Devlin et al. 2018)
Delerium assessment/prevention

Recommendation: Regular assessment of delirium using validated screening tools for critically ill adults.

Based on your expertise as a clinician, do you think this EBP is sufficiently important to include?

- Yes (1)
- Maybe (2)
- No (3)

4.30_Text Please comment on any additional thoughts about the above EBP below:

____

____

____

____

____

4.3
EBP 4.3 (Devlin et al. 2018)
Delerium assessment/prevention

Recommendation: Using noise and light reduction strategies to improve sleep in critically ill adults.

Based on your expertise as a clinician, do you think this EBP is sufficiently important to include?

- ○ Yes (1)
- ○ Maybe (2)
- ○ No (3)

4.3_Text Please comment on any additional thoughts about the above EBP below:

____

____

____

____

____

4.42
EBP 4.42 (Devlin et al. 2018)
[Merged with EBP 14.1 (Dammeyer et al. 2013)]
Early mobilization

Recommendation: Performing rehabilitation or mobilization in critically ill adults with mobility protocols executed by nurses and PTs with family involvement.

Based on your expertise as a clinician, do you think this EBP is sufficiently important to include?

- ◯ Yes (1)
- ◯ Maybe (2)
- ◯ No (3)

4.42_Text Please comment on any additional thoughts about the above EBP below:

____

____

____

____

____

15.2
EBP 15.2 (Murray 2016)
Neuromuscular blockade

Recommendation: Scheduled eye care that includes lubricating drops or gel and eyelid closure for patients receiving continuous neuromuscular-blockading agents.

Based on your expertise as a clinician, do you think this EBP is sufficiently important to include?

- ◯ Yes (1)
- ◯ Maybe (2)
- ◯ No (3)

15.2_Text Please comment on any additional thoughts about the above EBP below:

____

____

____

____

____

15.3
EBP 15.3 (Murray 2016)
Neuromuscular blockade

Recommendation: Neuromuscular-blocking agent administered by continuous intravenous infusion early in the course of acute respiratory distress syndrome for patients with a Pao2/Fio2 less than 150.

Based on your expertise as a clinician, do you think this EBP is sufficiently important to include?

○ Yes (1)

○ Maybe (2)

○ No (3)

15.3_Text Please comment on any additional thoughts about the above EBP below:

____

____

____

____

____

15.5
EBP 15.5 (Murray 2016)
Neuromuscular blockade

Recommendation: Use/trial of a neuromuscular-blocking agents in life-threatening situations associated with profound hypoxemia, respiratory acidosis, or hemodynamic compromise.

Based on your expertise as a clinician, do you think this EBP is sufficiently important to include?

- ○ Yes (1)
- ○ Maybe (2)
- ○ No (3)

15.5_Text Please comment on any additional thoughts about the above EBP below:

____

____

____

____

____

15.9
EBP 15.9 (Murray 2016)
Neuromuscular blockade

Recommendation: Patients receiving a continuous infusion of neuromuscular-blocking agent should receive a structured physiotherapy regimen.

Based on your expertise as a clinician, do you think this EBP is sufficiently important to include?

○ Yes (1)

○ Maybe (2)

○ No (3)

15.9_Text Please comment on any additional thoughts about the above EBP below:

____

____

____

____

____

15.10
EBP 15.10 (Murray 2016)
Neuromuscular blockade

Recommendation: Target blood glucose level for less than 180mg/dL in patients receiving neuromuscular-blocking agents.

Based on your expertise as a clinician, do you think this EBP is sufficiently important to include?

○ Yes (1)

○ Maybe (2)

○ No (3)

15.10_Text Please comment on any additional thoughts about the above EBP below:

____

____

____

____

____

2.1
EBP 2.1 (Chu et al. 2018)
Liberal oxygen therapy

Recommendation: Recommend against liberal oxygen therapy in acutely ill adults (aged ≥18 years) defined as saturation of peripheral oxygen [SpO_2] above range of 94% to 96%.

Based on your expertise as a clinician, do you think this EBP is sufficiently important to include?

- ○ Yes (1)
- ○ Maybe (2)
- ○ No (3)

2.1_Text Please comment on any additional thoughts about the above EBP below:

____

____

____

____

____

X→

18.1
EBP 18.1 (Lu 2017)
Open lung strategy

Recommendation: OLS (open lung strategy) during mechanical ventilation for patients with ARDS.

Based on your expertise as a clinician, do you think this EBP is sufficiently important to include?

- ○ Yes (1)
- ○ Maybe (2)
- ○ No (3)

18.1_Text Please comment on any additional thoughts about the above EBP below:

____

____

____

____

____

Page Break

5.3
EBP 5.3 (Girard et al. 2016)
Cuff leak test in high risk patients

Recommendation: Perform cuff leak test in MV adults who meet extubation criteria and are deemed high risk for post-extubation stridor

Based on your expertise as a clinician, do you think this EBP is sufficiently important to include?

- ◯ Yes (1)
- ◯ Maybe (2)
- ◯ No (3)

5.3_Text Please comment on any additional thoughts about the above EBP below:

____
____
____
____
____

5.1
EBP 5.1 (Girard et al. 2016)
Early mobilization

Recommendation: For acutely hospitalized patients who have ben mechanically ventilated for >24h, suggest protocolized rehabilitation directed toward early mobilization.

Based on your expertise as a clinician, do you think this EBP is sufficiently important to include?

○ Yes (1)

○ Maybe (2)

○ No (3)

5.1_Text Please comment on any additional thoughts about the above EBP below:

____

____

____

____

____

X→ X→

5.2
EBP 5.2 (Girard et al. 2016)
Ventilator liberation protocol

Recommendation: Manage acutely hosp adults who have been MV >24 hours with a ventilator liberation protocol (designed to reduce variation in practice).

Based on your expertise as a clinician, do you think this EBP is sufficiently important to include?

○ Yes (1)

○ Maybe (2)

○ No (3)

5.2_Text Please comment on any additional thoughts about the above EBP below:

____

____

____

____

____

5.4
EBP 5.4 (Girard et al. 2016)
[Merged with EBP 3.7 (Schmidt et al. 2016)]
Short term steroids for high risk patients who fail cuff leak test

Recommendation: For adults who have failed cuff leak test but are otherwise ready for extubation, administer systemic steroids for at least 4 hours before extubation

Based on your expertise as a clinician, do you think this EBP is sufficiently important to include?

- ○ Yes (1)
- ○ Maybe (2)
- ○ No (3)

5.4_Text Please comment on any additional thoughts about the above EBP below:

____

____

____

____

____

6.3
EBP 6.3 (Ouellette et al. 2017)
Extubation

Recommendation: Extubation to preventive non-invasive ventilation (NIV).

Based on your expertise as a clinician, do you think this EBP is sufficiently important to include?

- ○ Yes (1)
- ○ Maybe (2)
- ○ No (3)

6.3_Text Please comment on any additional thoughts about the above EBP below:

____
____
____
____
____

Page Break

MNG_Hernandez
Hernandez 2016

Recommendation: Extubation to high flow nasal cannula.

Based on your expertise as a clinician, do you think this EBP is sufficiently important to include?

- Yes (1)
- Maybe (2)
- No (3)

MNG_Hernandez_Text Please comment on any additional thoughts about the above EBP below:

____

____

____

____

____

TH_Weidemann
Weidemann 2016

Recommendation: Conservative fluid management

Based on your expertise as a clinician, do you think this EBP is sufficiently important to include?

- ◯ Yes (1)
- ◯ Maybe (2)
- ◯ No (3)

TH_Weidemann_Text Please comment on any additional thoughts about the above EBP below:

____

____

____

____

____

TH_Strom
Strom 2010
Recommendation: Analgesia-first approach

Based on your expertise as a clinician, do you think this EBP is sufficiently important to include?

- ◯ Yes (1)
- ◯ Maybe (2)
- ◯ No (3)

TH_Strom_Text Please comment on any additional thoughts about the above EBP below:

____

____

____

____

____

TH_Girard
Girard 2008

Recommendation: Paired spontaneous awakening trials (SATs) and spontaneous breathing trials (SBTs)

Based on your expertise as a clinician, do you think this EBP is sufficiently important to include?

- ◯ Yes (1)
- ◯ Maybe (2)
- ◯ No (3)

TH_Girard_Text Please comment on any additional thoughts about the above EBP below:

____

____

____

____

____

Page Break

Final_Text Please leave any other comments or thoughts you have, below:

____

____

____

____

____

End of Block: Default Question Block

DIGITAL-C Site EBP Survey (Consolidated)

Start of Block: Intro

Intro We would like you to complete this survey in order to help us prioritize potential evidence based practices (EBPs) to focus on. These initial EBPs have been identified from a review of guidelines and recommendations in the literature.

This project has been deemed exempt by the University of Michigan Institutional Review Board, HUM00149237.

Name Name

____

Inst Institution

____

Role Please indicate your role (e.g. physician, nurse, RT)

____

ICUType Please list the type of ICU you practice in (e.g. medical, surgical, etc.)

____

GLIA Defs We ask you to assess each EBP according to the criteria below, which are adapted from the GLIA* instrument: **Measurable**- Endpoints or markers are well identified in this EBP to make it easily measurable and computable in the EHR. **Resource Intensive**- The EBP is resource intensive. **Credible author**- The organizations and authors who developed the guideline have credibility with the intended audience of the guideline.
*GuideLine Implementability Appraisal (GLIA)- https://doi.org/10.1186/1472-6947-5-23

End of Block: Intro

Start of Block: Phase 1

X→ X→

1.1
EBP 1.1 (Fan et al. 2017)
Lung Protective Ventilation

Recommendation: Mechanical ventilation using lower tidal volumes (4–8 ml/kg predicted body weight) and lower inspiratory pressures (plateau pressure < 30 cm H_2O) for adult patients with ARDS

Based on your expertise as a clinician, do you think this EBP is sufficiently important to include?

- ○ Yes (1)
- ○ Maybe (2)
- ○ No (3)

1.1_GLIA Please rank the degree to which you agree that the criteria below apply to the EBP above.

	1-Highly Disagree (1)	2 (2)	3 (3)	4 (4)	5- Highly Agree (5)
Measurable (1)	○	○	○	○	○
Resource Intensive (3)	○	○	○	○	○
Credible Author (2)	○	○	○	○	○

1.1_Text Please comment on any additional thoughts about the above EBP below:

____

____

____

____

____

End of Block: Phase 1

Start of Block: Phase 2

X→ X→

2.1
EBP 2.1 (Devlin et al. 2018; Ouellette et al. 2017)
Sedation protocols

Recommendation: Using either propofol or dexmedetomidine is preffered over benzodiazepines for sedation in critically ill, mechanically ventilated adults

Based on your expertise as a clinician, do you think this EBP is sufficiently important to include?

- ○ Yes (1)
- ○ Maybe (2)
- ○ No (3)

2.1_GLIA Please rank the degree to which you agree that the criteria below apply to the EBP above.

	1-Highly Disagree (1)	2 (2)	3 (3)	4 (4)	5- Highly Agree (5)
Measurable (1)	○	○	○	○	○
Resource Intensive (3)	○	○	○	○	○
Credible Author (2)	○	○	○	○	○

2.1_Text Please comment on any additional thoughts about the above EBP below:

____

____

____

____

____

2.2
EBP 2.2 (Fan et al. 2017)

Prone Positioning

Recommendation: Prone positioning for more than 12 hours/day for adult patients with severe ARDS.

Based on your expertise as a clinician, do you think this EBP is sufficiently important to include?

- ○ Yes (1)
- ○ Maybe (2)
- ○ No (3)

2.2_GLIA Please rank the degree to which you agree that the criteria below apply to the EBP above.

	1-Highly Disagree (1)	2 (2)	3 (3)	4 (4)	5- Highly Agree (5)
Measurable (1)	○	○	○	○	○
Resource Intensive (3)	○	○	○	○	○
Credible Author (2)	○	○	○	○	○

2.2_Text Please comment on any additional thoughts about the above EBP below:

____

____

____

____

____

2.3
EBP 2.3 (Devlin et al. 2018, Ouelette et al. 2017; Schmidt et al. 2016; Girard et al. 2016)
Daily awakening and breathing trials

Recommendation: Use of daily sedative interruption (DSI)/nurse-protocolized sedation to achieve and maintain a light level of sedation

Based on your expertise as a clinician, do you think this EBP is sufficiently important to include?

- Yes (1)
- Maybe (2)
- No (3)

2.3_GLIA Please rank the degree to which you agree that the criteria below apply to the EBP above.

	1-Highly Disagree (1)	2 (2)	3 (3)	4 (4)	5- Highly Agree (5)
Measurable (1)	○	○	○	○	○
Resource Intensive (3)	○	○	○	○	○
Credible Author (2)	○	○	○	○	○

2.3_Text Please comment on any additional thoughts about the above EBP below:

____

____

____

____

____

2.4
EBP 2.4 (Devlin et al. 2018)
Protocol-based pain assessment and management

Recommendation: Routine use of an assessment-driven, protocol-based, stepwise approach for pain and sedation management in critically ill adults. Pain should be treated before a sedative agent is considered.

Based on your expertise as a clinician, do you think this EBP is sufficiently important to include?

- ○ Yes (1)
- ○ Maybe (2)
- ○ No (3)

2.4_GLIA Please rank the degree to which you agree that the criteria below apply to the EBP above.

	1-Highly Disagree (1)	2 (2)	3 (3)	4 (4)	5- Highly Agree (5)
Measurable (1)	○	○	○	○	○
Resource Intensive (3)	○	○	○	○	○
Credible Author (2)	○	○	○	○	○

2.4_Text Please comment on any additional thoughts about the above EBP below:

____

____

____

____

____

2.5
EBP 2.5 (Devlin et al. 2018; Dammeyer et al. 2013; Girard et al. 2016)
Early mobilization

Recommendation: Performing rehabilitation or mobilization in critically ill adults with mobility protocols executed by nurses and PTs with family involvement.

Based on your expertise as a clinician, do you think this EBP is sufficiently important to include?

○ Yes (1)

○ Maybe (2)

○ No (3)

2.5_GLIA Please rank the degree to which you agree that the criteria below apply to the EBP above.

	1-Highly Disagree (1)	2 (2)	3 (3)	4 (4)	5- Highly Agree (5)
Measurable (1)	○	○	○	○	○
Resource Intensive (3)	○	○	○	○	○
Credible Author (2)	○	○	○	○	○

2.5_Text Please comment on any additional thoughts about the above EBP below:

____

____

____

____

____

2.6
EBP 2.6 (Wiedemann et al,, 2006; Silversides et al., 2017)
Conservative fluid management

Recommendation: Conservative fluid management

Use of a conservative approach to fluid administration or deresuscitation following haemodynamic stabilisation of critically ill patients; restricted fluid intake and increased urinary output.
Based on your expertise as a clinician, do you think this EBP is sufficiently important to include?

○ Yes (1)

○ Maybe (2)

○ No (3)

2.6_GLIA Please rank the degree to which you agree that the criteria below apply to the EBP above.

	1-Highly Disagree (1)	2 (2)	3 (3)	4 (4)	5- Highly Agree (5)
Measurable (1)	○	○	○	○	○
Resource Intensive (3)	○	○	○	○	○
Credible Author (2)	○	○	○	○	○

2.6_Text Please comment on any additional thoughts about the above EBP below:

____

____

____

____

____

2.7
EBP 2.7 (Strom et al. 2010)
Analgesia-first approach to sedation

Recommendation: Strategy to keep the amount and duration of sedation to a minimum

Based on your expertise as a clinician, do you think this EBP is sufficiently important to include?

○ Yes (1)

○ Maybe (2)

○ No (3)

2.7_GLIA Please rank the degree to which you agree that the criteria below apply to the EBP above.

	1-Highly Disagree (1)	2 (2)	3 (3)	4 (4)	5- Highly Agree (5)
Measurable (1)	○	○	○	○	○
Resource Intensive (3)	○	○	○	○	○
Credible Author (2)	○	○	○	○	○

2.7_Text Please comment on any additional thoughts about the above EBP below:

____

____

____

____

____

End of Block: Phase 2

Start of Block: Phase 3

3.1
EBP 3.1 (Girard et al. 2016)
Ventilator liberation protocol

Recommendation: Manage acutely hosp adults who have been MV >24 hours with a ventilator liberation protocol (designed to reduce variation in practice).

Based on your expertise as a clinician, do you think this EBP is sufficiently important to include?

- ◯ Yes (1)
- ◯ Maybe (2)
- ◯ No (3)

3.1_GLIA Please rank the degree to which you agree that the criteria below apply to the EBP above.

	1-Highly Disagree (1)	2 (2)	3 (3)	4 (4)	5- Highly Agree (5)
Measurable (1)	◯	◯	◯	◯	◯
Resource Intensive (3)	◯	◯	◯	◯	◯
Credible Author (2)	◯	◯	◯	◯	◯

3.1_Text Please comment on any additional thoughts about the above EBP below:

____

____

____

____

____

3.2
EBP 3.2 (Ouellette et al. 2017)
Recommendation: Extubation to preventive non-invasive ventilation (NIV).

Based on your expertise as a clinician, do you think this EBP is sufficiently important to include?

○ Yes (1)

○ Maybe (2)

○ No (3)

3.2_GLIA Please rank the degree to which you agree that the criteria below apply to the EBP above.

	1-Highly Disagree (1)	2 (2)	3 (3)	4 (4)	5- Highly Agree (5)
Measurable (1)	○	○	○	○	○
Resource Intensive (3)	○	○	○	○	○
Credible Author (2)	○	○	○	○	○

3.2_Text Please comment on any additional thoughts about the above EBP below:

____

____

____

____

____

3.3
EBP 3.2 (Hernandez et al. 2016)

Recommendation: Extubation to high flow nasal cannula.

Based on your expertise as a clinician, do you think this EBP is sufficiently important to include?

○ Yes (1)

○ Maybe (2)

○ No (3)

3.3_GLIA Please rank the degree to which you agree that the criteria below apply to the EBP above.

	1-Highly Disagree (1)	2 (2)	3 (3)	4 (4)	5- Highly Agree (5)
Measurable (1)	○	○	○	○	○
Resource Intensive (3)	○	○	○	○	○
Credible Author (2)	○	○	○	○	○

3.3_Text Please comment on any additional thoughts about the above EBP below:

____

____

____

____

____

End of Block: Phase 3

Start of Block: Final Comments

Final_Text Please leave any other comments or thoughts you have, including suggestions for other EBPs to include or feedback on the survey:

____

____

____

____

____

End of Block: Final Comments
